# Virtual and in Vitro Screening Employing a Repurposing Approach Reveal 13‐*cis*‐Retinoic Acid is a PTP1B Inhibitor

**DOI:** 10.1002/cmdc.202400452

**Published:** 2024-10-08

**Authors:** Reyna Del Carmen Navarrete‐Mondragón, Francisco Cortés‐Benítez, Jessica Elena Mendieta‐Wejebe, Martin González‐Andrade, Jaime Pérez‐Villanueva

**Affiliations:** ^1^ Doctorado en Ciencias Biológicas y de la Salud Universidad Autónoma Metropolitana [1 Ciudad de México 04960 México; ^2^ Departamento de Sistemas Biológicos División de Ciencias Biológicas y de la Salud Universidad Autónoma Metropolitana-Xochimilco [1 Ciudad de México 04960 México; ^3^ Laboratorio de Biofísica y Biocatálisis Sección de Estudios de Posgrado e Investigación Escuela Superior de Medicina Instituto Politécnico Nacional Plan de San Luis y Salvador Díaz Mirón s/n, Casco de Santo Tomás, Miguel Hidalgo Ciudad de México 11340 México; ^4^ Departamento de Bioquímica Facultad de Medicina Universidad Nacional Autónoma de México Ciudad de México 04510 México

**Keywords:** Type 2 diabetes (T2D), Protein tyrosine phosphatase 1B (PTP1B), Virtual screening, 13-*cis*-Retinoic acid, Drug repositioning, Polypharmacology

## Abstract

Current treatments for type 2 diabetes (T2D) mainly rely on exercise, dietary control, and anti‐diabetic drugs to enhance insulin secretion and improve insulin sensitivity. However, there is a need for more therapeutic options, as approved drugs targeting different pharmacological objectives are still unavailable. One potential target that has attracted attention is the protein tyrosine phosphatase 1B (PTP1B), which negatively regulates the insulin signaling pathway. In this work, a comprehensive computational screening was carried out using cheminformatics and molecular docking on PTP1B, employing a rigorous repurposing approach. The screening involved approved drugs and compounds under research as anti‐diabetics that bind to targets such as peroxisome proliferator‐activated receptor gamma (PPAR‐γ) and α‐glucosidase. Several computational hits were then meticulously tested in vitro against PTP1B, with 13‐*cis*‐retinoic acid (**3a**) showing an IC_50_ of 0.044 mM and competitive inhibition. Molecular dynamics studies further confirmed that **3a** can bind to the catalytic binding site of PTP1B. Finally, **3a** is the first time it has been reported as an inhibitor of PTP1B, making it a potentially valuable candidate for further studies in D2T treatment.

## Graphical abstract

A computational screening using cheminformatics and molecular docking has been performed on PTP1B. *In vitro* assays have revealed that 13‐*cis*‐retinoic acid is a competitive inhibitor. This is the first report of 13‐*cis*‐retinoic acid as PTP1B inhibitor, making it a valuable candidate for future research toward its repositioning.

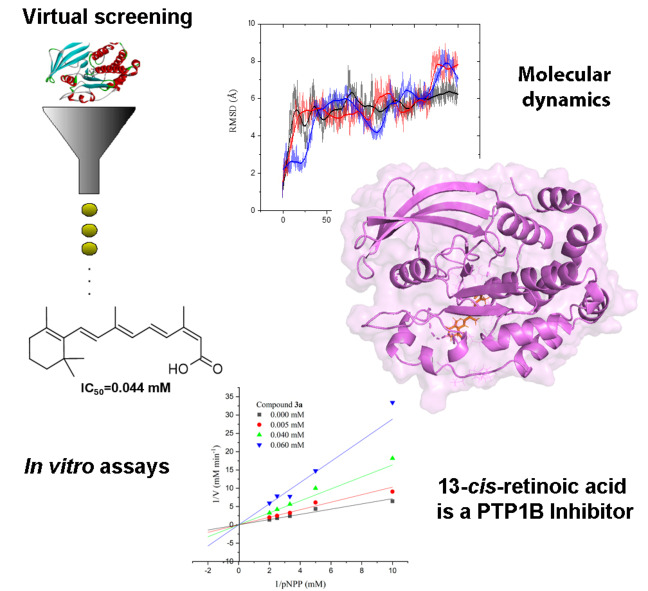

## 1 Introduction

Diabetes is a group of metabolic diseases that cause high blood glucose levels (hyperglycemia). This happens due to a lack of insulin in the case of type 1 diabetes (T1D) or defects in insulin action and secretion in the case of type 2 diabetes (T2D).[^1^, ^2^, ^3^, ^4^, ^5^] In 2021, the International Diabetes Federation reported that 425 million people worldwide had diabetes, with the majority having T2D. Unfortunately, this number is expected to increase to 783 million by 2045, posing a significant challenge to healthcare organizations.[^3^, ^6^] The current classification of diabetes based primarily on aetiology includes mainly T1D, T2D, gestational diabetes and other specific types of diabetes.[^7^, ^8^, ^9^, ^10^] T2D is the most frequent, comprising 90–95 % of cases. It is due to a continuous loss of insulin secretion, usually with insulin resistance.^[11]^ Insulin is a critically important hormone that controls glucose homeostasis and other cellular activities such as protein synthesis, gene transcription and substrate metabolism.^[11]^

**Figure 1:**
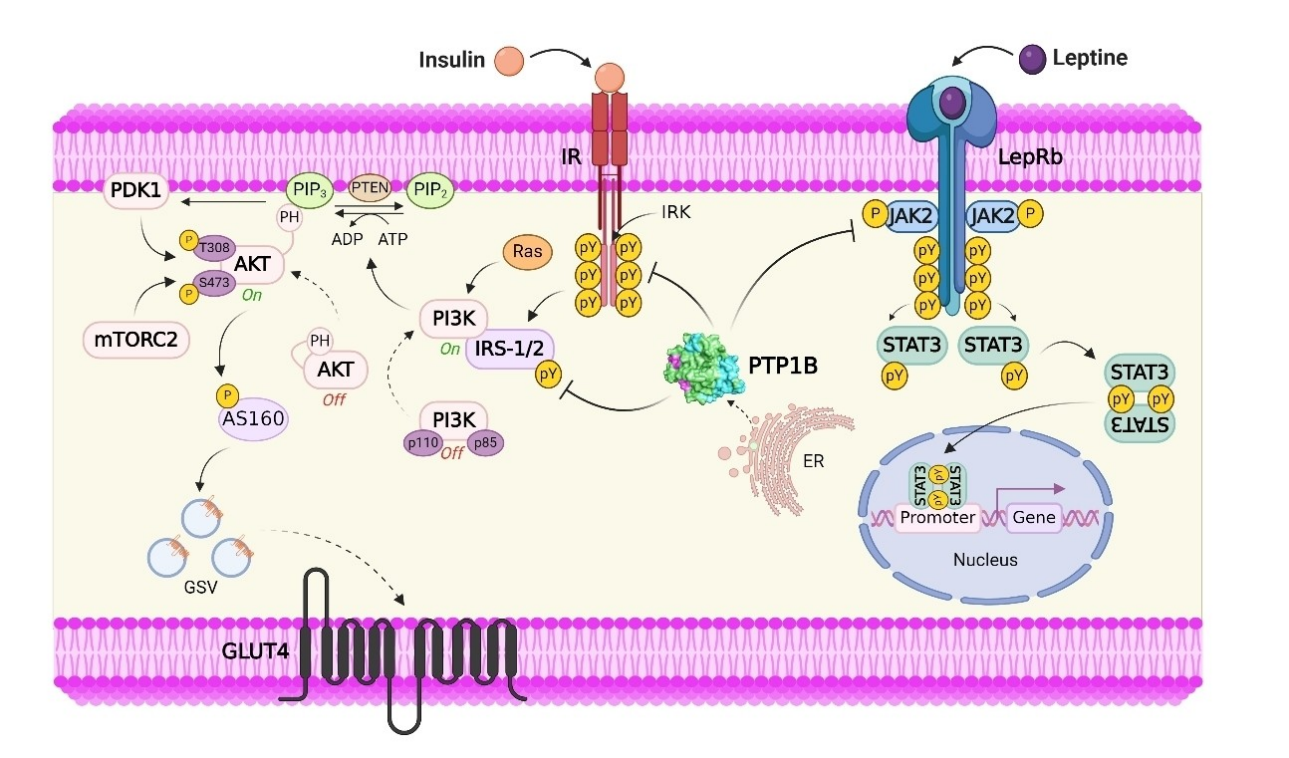
Metabolic signaling pathways modulated by PTP1B. PTP1B acts as a negative regulator of insulin and leptin signaling pathways. In the insulin pathway, PTP1B dephosphorylates tyrosine residues in IR, IRS‐1 and IRS‐2, supporting glucose homeostasis by modulating GLUT4 transit. In the leptin signaling pathway, PTP1B dephosphorylates LepRb and JAK2, inactivating STAT3 and thus controlling the expression of genes (POMC and SOCS_3_) involved in energy balance. Abbreviature: ADP: adenosine diphosphate. ATP: adenosine triphosphate. Akt: Ak strain transforming. AS160: Akt substrate of 160 kDa. ER: endoplasmic reticulum. GLUT4: glucose transporter type 4. GSV: glucose storage vesicles. IR: insulin receptor. IRK: tyrosine kinase domain of the insulin receptor. IRS‐1/2: insulin receptor substrate 1/2 . JAK2: Janus kinase 2. LepRb: short isoform of the leptin receptor. mTORC2: mammalian/mechanistic target of rapamycin (mTOR) complex 2. PDK1: phosphatidylinositol‐dependent kinase 1. PH: pleckstrin homology domain. PI3 K: Phosphoinositide 3‐kinases. PIP2: Phosphatidylinositol 4,5‐bisphosphate. PIP3: phosphatidylinositol 3,4,5 trisphosphate. POMC: proopiomelanocortin. PTEN: phosphatase and tensin homolog. PTP1B: protein tyrosine phosphatase 1B. pY: phosphotyrosine. Ras: rat sarcoma virus. SOCS_3_: suppressor of cytokine signaling 3. STAT3: signal transducer and activator of transcription 3.

**Figure 2:**
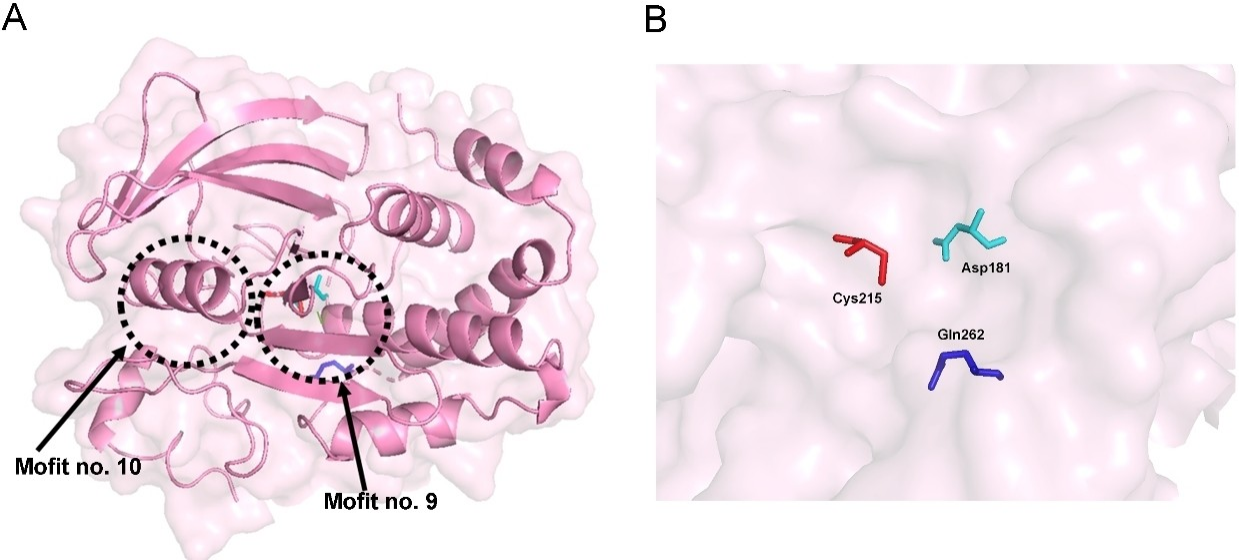
Schematic illustration of the 3D structure of PTP1B. (A) Representation of the PTP1B enzyme. The PTP1B enzyme harbors 10 clearly identifiable motifs. The 3D structure of PTP1B is shown with the motifs critical for the enzymatic reaction indicated in different colors. (B) A bar model illustrates the critical catalytic residues in the secondary structure: Cys 215 (red), Asp 181 (cyan) and Gln 262 (blue).

Treatment of T2D includes dietary control, exercise, and the use of drugs to increase insulin secretion and improve insulin sensitivity.^[12]^ Currently, different types of oral hypoglycemic drugs are available for the treatment of patients with T2D.^[13]^ However, it has been shown that hypoglycemic agents have certain limitations and cause side effects, some of which are weight gain, gastrointestinal problems, headache, peripheral edema, and hypotension.^[10]^ It is important to find new approaches to combat chronic diseases such as T2D because current therapies can sometimes be ineffective due to drug resistance. This global problem highlights the need for more effective drugs with different mechanisms of action. In recent years, protein tyrosine phosphatase 1B (PTP1B) has attracted attention as a potential therapeutic target for developing new drugs, especially for treating T2D and obesity.[^13^, ^14^, ^15^, ^16^] Since it is a negative regulator of the insulin signaling pathway, also PTP1B has been shown to play an important role in metabolic signaling (especially obesity and T2D) through interaction with the IR and JAK2 downstream of the leptin receptor. PTP1B acts as a negative regulator of insulin and leptin signaling pathways. In the insulin signaling pathway, PTP1B dephosphorylates phosphotyrosine residues on insulin receptor (IR), insulin receptor substrate‐1 (IRS‐1) and insulin receptor substrate‐2 (IRS‐2). This action promotes controlled glucose uptake into the cell by regulating the transit of Glucose Transporter Type 4 (GLUT4) from the cytoplasm to the cell membrane. In the leptin signaling pathway,^[13]^ PTP1B helps maintain energy balance. It does so by inactivating Signal Transducer and Activator of Transcription 3 (STAT3) and its target genes (POMC and SOCS_3_) after dephosphorylating the Leptin Receptor (LepRb) and Janus Kinase 2 (JAK2) (Figure 1). There is evidence linking PTP1B to the ER stress response through interaction with PERK1B, although the effect of this interaction is still debated. PTP1B can also influence inflammation by regulating the various JAKs and STATs, suggesting an interesting and still largely undiscovered role in immune signalling. The PTP1B enzyme harbors 10 identifiable motifs. Motif no. 9 contains the Cys 215 residue of the catalytic site, as well as Asp 181 and Gln 262 (Figure 2).^[17]^

In the search for new drugs, there is a variety of strategies, one of the most interesting is drug repositioning.^[18]^ Overall, drug repositioning consists of reusing an active pharmaceutical ingredient already on the market for other therapeutic purposes. This strategy has the advantage that since these are compounds with clinical application or in development, their properties, pharmacokinetics, and toxicology are also known to a large extent, thus saving time and resources.^[19]^ New computational methods based on data mining have been developed to identify new candidates for drug repurposing, giving new opportunities to find new applications for known compounds, in this case, for treating T2D. ^[20]^

Therefore, the present work focuses on the search for drugs with anti‐diabetic activity using a repositioning strategy.^[21]^ To do so, a virtual screening study was performed on a database of CHEMBL drugs and compounds with known activity against other anti‐diabetic targets, such as PPAR‐γ and α‐glucosidase.^[22]^ The database was narrowed down to compounds with shared chemotypes with PTP1B inhibitors, and the resulting compounds were subsequently subjected to molecular docking. Among the best candidates, a representative sample of compounds were chosen to be evaluated; eight structures derived from screening against PTP1B with code 1C83, and six structures for derived from screening against PTP1B with code 1T49. *In vitro* assays revealed that 13‐*cis*‐retinoic acid is a competitive PTP1B inhibitor. Although, 13‐*cis*‐retinoic acid has been previously reported for different applications, herein it is reported as PTP1B inhibitor of by the first time.

## 2 Results and Discussion

### 2.1 Databases

The reference database consisted of 4285 compounds with activity against PTP1B; after cleaning and applying the inclusion criteria, 2673 compounds with IC_50_ values≤100 μM were obtained. The same criteria were applied to compounds from the search database, including 605 α‐glucosidase inhibitors, 2135 PPARγ ligands and 10849 approved drugs; the same resulted after the application of the criteria in 524 α‐glucosidase inhibitors, 1665 PPARγ ligands and 7621 drugs, giving a total of 9810 compounds for the refined search database. The latter database was formulated to search compounds for drug repositioning purposes and with possible anti‐diabetic effects via a polypharmacological profile. Both databases were classified into chemotypes using the *Bemis‐Murcko* system.^[23]^

**Figure 3:**
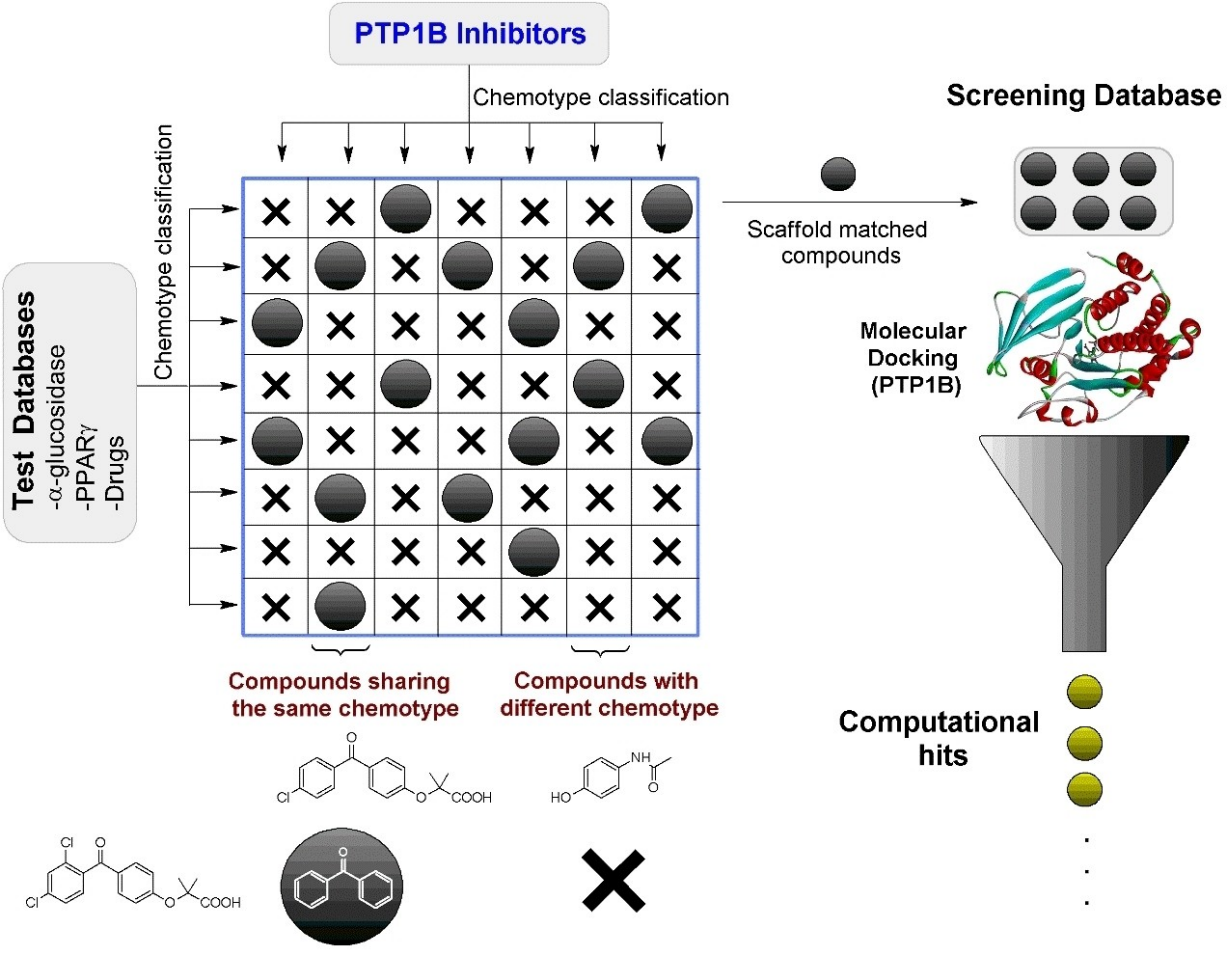
Methodological design for computational screening of compounds on PTP1B.

Examples of the most frequent chemotypes can be found in the supplementary material (Figure S1), for the database of compounds active against PTP1B, 1091 different chemotypes were found. A total of 5448 chemotypes were retrieved from the search database. As some of these chemotypes were already present in the database of active compounds against PTP1B, a selection was made to include only those compounds that share chemotypes with the active compounds (IC_50_≤100 μM). This resulted in 1075 compounds used for molecular docking (Figure 3).

### 2.2 Structural Model of PTP1B_1–400_

**Figure 4:**
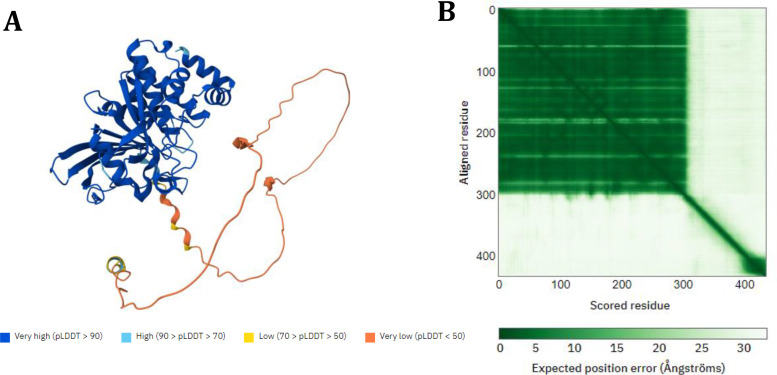
Prediction results of the complete structural model of PTP1B_1–400_. A) AlphaFold produces a per‐residue model confidence score (pLDDT) and B) Predicted aligned error (PAE).

The structural model of the hPTP1B_1–400_ protein was obtained from AlphaFold Protein Structure Database developed by DeepMind and EMBL‐EBI (https://alphafold.ebi.ac.uk/).^[24]^ The pdb file was downloaded from the following link: https://alphafold.ebi.ac.uk/entry/P18031. This structure was subjected to a molecular dynamics simulation for 200 ns to find the most stable conformation of the unstructured region (301–400). The AlphaFold structural model shows a very good confidence score for the region 1–300 amino acids and very low for the region from 300–400, which corresponds to the intrinsically unstructured region of the enzyme (Figure 4). It is to be expected that prediction through artificial intelligence cannot resolve this last region since these intrinsically disordered structures tend to adopt multiple conformational options, as has been demonstrated in previous work.^[25]^

**Figure 5:**
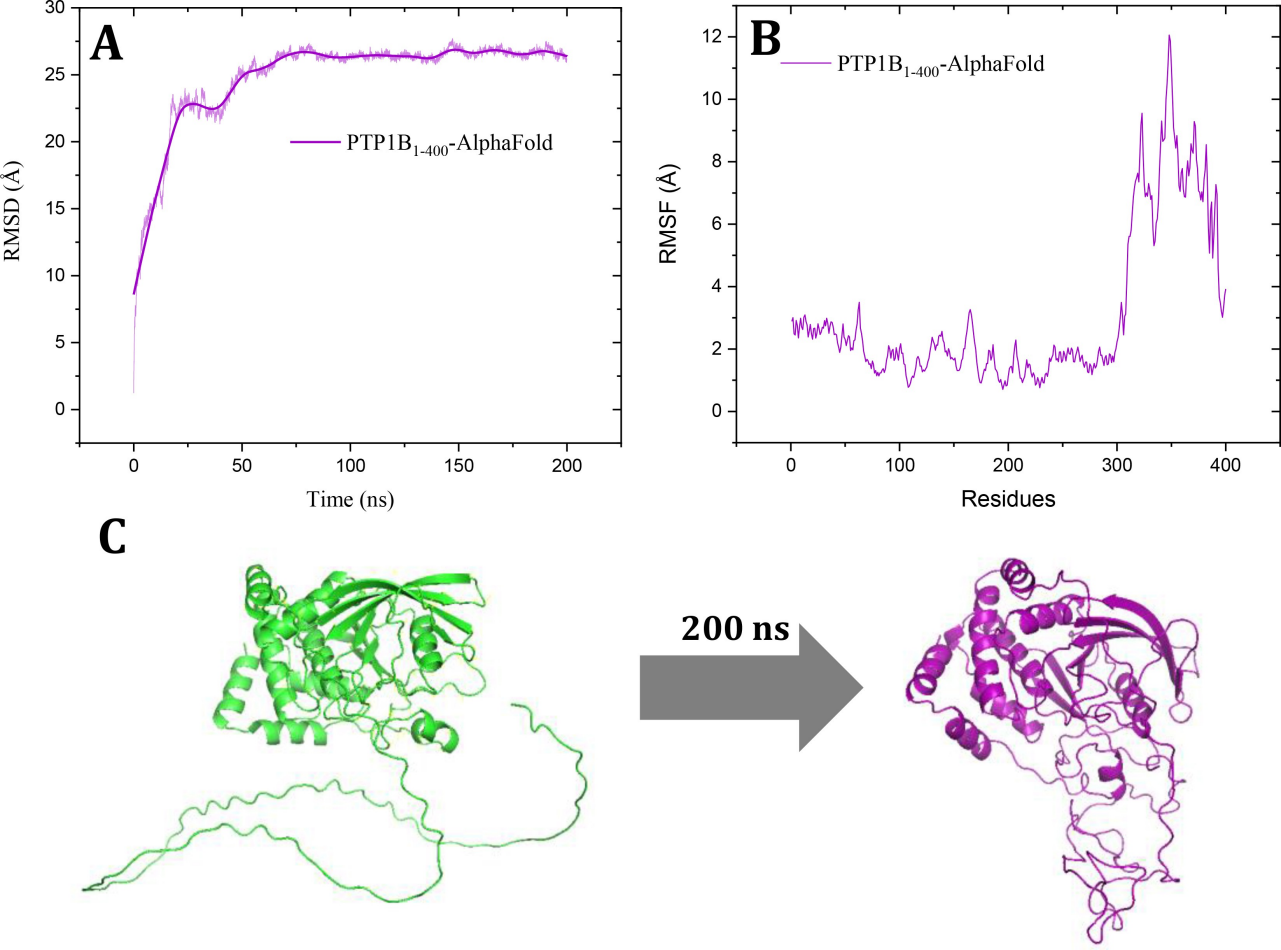
Analysis of 200 ns of molecular dynamics simulation. **A**) The RMSD & Time plot, **B**) The RMSF & Residues plot, and **C**) Structural model at the beginning and after 200 ns of the molecular dynamics of the PTP1B_1–400_.

With the aim of obtaining a complete three‐dimensional model of PTP1B, which would have biological relevance and could be used in theoretical studies, we subjected the model obtained from AlphaFold to MDS for 200 ns (video S1). Figure 5 shows the results obtained, where we can highlight that from 70 ns onwards the RMSD remains stable around 25 Å; regarding the RMSF, it is observed that the region from 300–400 is the one that fluctuates the most since this is the one that is folding along the MDS. Panel C of Figure 5 shows the beginning and end of MDS, where we can see that the catalytic region does not have significant structural changes, while the intrinsically unstructured region folds adopting a more stable conformation.

### 2.3 Molecular Docking

We conducted molecular docking studies using the X‐ray structures of PTP1B (PDB ID: 1C83 and 1T49).[^26^, ^27^] We used three programs: Vina, Autodock (included in YASARA structure suite), and GOLD for the docking calculations. Since different programs employ different functions to afford docking results, it is important to use different software to access a more reliable consensus screening.^[28]^

First, we validated the method for the two crystallographic structures by re‐docking the co‐crystal structure of compound **OAI** at the catalytic site of PTP1B (PDB ID: 1C83) and compound **892** to the allosteric site of this enzyme (PDB ID: 1T49).[^29^, ^30^] Then, we calculated the root mean square deviation (RMSD) value between the position of the ligands resulting from the docking and the original position in the crystallographic structure, as shown in Table 1, similarly performed for the complete PTP1B_1–400_ structure.^[30]^ The RMSD values were less than 2 Å.^[31]^

**Table 1:** Validation of docking of 1C83 (OAI), 1T49 (892) co‐crystallized and PTP1B_1–400_ structures.

Protein	Program	Score*	RMSD (Å)
**1C83 (OAI)**	AutoDock 4.2	−9.92	0.0
	AutoDock VINA	−8.10	0.74
	GOLD	55.25	0.77
**1T49 (892)**	AutoDock 4.2	−11.86	0.79
	AutoDock VINA	−8.90	0.66
	GOLD	60.30	1.06
PTP1B_1–400_ **(OAI)**	AutoDock 4.2	−9.60	0.3
	AutoDock VINA	−9.03	0.63
	GOLD	65.45	0.73
PTP1B_1–400_ **(892)**	AutoDock 4.2	−10.93	0.82
	AutoDock VINA	−9.83	0.76
	GOLD	74.63	1.03

[a] AutoDock Vina and 4.2 is expressed as ΔG in kcal/mol; Gold is expressed as ChemPLP fitness score.

To obtain the compounds with the lowest binding energy *in silico*: the VSdatabase was subjected to virtual screening^[32]^ by consecutively employing Autodock Vina,[^33^, ^34^, ^35^] Autodock 4.2 (both included YASARA structure software),^[36]^ and GOLD.[^27^, ^37^] This study aimed to determine the most accurate solution for each ligand‐protein interaction by combining information from various scoring functions. Initially, Vina was used to sort the results based on the minimum energy pose score. The results were then divided into four categories, which were quartiles Q1–Q4. Only the best Autodock Vina results (Q1, 268 compounds) were used in the following Autodock 4.2 and GOLD docking stages.^[28]^ This was done to reduce the calculation time and optimize the virtual screening process.

Then, 268 compounds were analyzed, and those with the lowest binding energy or best score were chosen from the three programs. For practical purposes, the results of the three programs were normalized from 0–1, where 0 is the compound with the highest binding energy, and 1 has the lowest *in silico* energy, respectively. Fifty compounds with the best scoring results were analyzed for the three programs (Tables S1 and S2). According to the results obtained, a representative series of 8 molecules [retrieved by docking against PTP1B (PDB ID:1C83)] was selected considering their commercial availability or easy synthetic accessibility: **1a** (camostat), **2a** (NCX 4016), **3a** (13‐*cis*‐Retinoic acid), **4a** (tolcapone), **5a** (estrone), **6a** (xelalipin), **7a** (estradiol) and **8a** (fenofibric acid), (Table 2). For PTP1B (PDB ID:1T49), six compounds were selected: **1b** (fenofibrate), **2b** (congo red free acid), **3b** (mestranol), **4b** (17‐(1‐Propynyl)‐17beta‐estradiol), **5b** (ethinyl estradiol) and **7a** (estradiol) (Table 3). These same hits were docked with the complete PTP1B structure (PTP1B_1–400_). Although, docking results employing PTP1B_1–400_ are similar to 1C83 and 1T49. It is important mentioning that **3a** showed better scores with all employing programs against PTP1B_1–400_ as compared to 1C83. The same trend was observed for **3b**, **4b** and **5b** as compared to 1T49 results.

**Table 2:** Compounds selected for biological assays as potential PTP1B inhibitors (catalytic site).

Compound	Structure	AD 4.2 (kcal/mol)		AD VINA (kcal/mol)		GOLD fitness (ChemPLP)	
		1C83	1–400	1C83	1–400	1C83	1–400
**1a** (camostat)	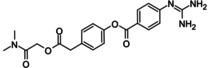	−9.95	−9.71	−8.97	−9.96	39.89	42.31
**2a** (NCX 4016)	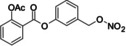	−10.52	−9.56	−8.65	−7.80	35.82	32.21
**3a** (13‐*cis*‐retinoic acid)	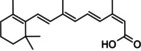	−8.06	−8.93	−8.28	−8.98	25.90	26.96
**4a** (tolcapone)	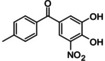	−9.63	−8.73	−8.14	−9.26	30.38	31.18
**5a** (estrone)	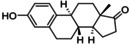	−9.24	−6.63	−8.22	−6.73	27.26	21.62
**6a** (xenalipin)	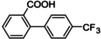	−8.81	−8.01	−7.89	−9.03	25.76	27.42
**7a** (estradiol)	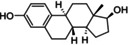	−8.82	−7.61	−8.62	−8.19	32.49	31.39
**8a** (fenofibric acid)	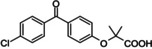	−8.85	−6.76	−7.86	−6.87	35.78	26.75

**Table 3:** Compounds selected for biological assays as potential PTP1B inhibitors (allosteric site).

Compound	Structure	AD 4.2 (kcal/mol)		AD VINA (kcal/mol)		GOLD fitness (ChemPLP)	
		1T49	1–400	1T49	1–400	1T49	1–400
**1b** (fenofibrate)	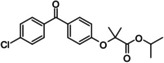	−7.68	−6.90	−8.48	−8.13	27.95	26.92
**2b** (congo red)	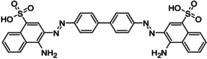	−11.28	−8.06	−11.32	−11.7	56.45	53.42
**3b** (mestranol)	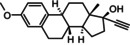	−8.17	−8.26	−6.55	−9.69	27.37	39.13
**4b** (17‐(1‐Propynyl)‐17beta‐estradiol)	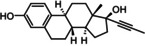	−8.37	−8.62	−7.34	−10.19	28.01	35.19
**5b** (ethinyl estradiol)	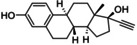	−8.17	−8.54	−8.46	−9.69	22.37	36.32
**7a** (estradiol)	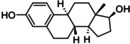	−8.23	−8.13	−8.53	−9.43	25.32	27.17

Overall, some interactions in common with the ligands in the crystallographic structure were observed for the computational hits. For the catalytic site (PDB ID: 1C83), hydrogen bonds interactions were observed with amino acids Lys^120^, Ser^216^, Arg^221^, Ala^217^ and Gly^220^; in addition, some alkyl, and pi‐alkyl interactions with Tyr^46^, Val^49^ and Phe^182^.^[29]^ On the other hand, for the allosteric site (PDB ID: 1T49) some salt bridge interactions were found with amino acids Ile^281^, Glu^276^, Asn^193^, others of alkyl and pi‐alkyl with Leu^192^ and Phe^280^ which correspond for the reported amino acids.^[17]^ It is essential to mention that the crystallographic ligand interactions at the catalytic site in the 1C83 structure correspond to Tyr^46^, Arg^45^, Asp^48^, Lys^120^, Phe^182^, Ile^219^, Asp^181^, and Gln^262^. In contrast, for the allosteric site, they are: Leu^192^, Asn^193^, Phe^196^, Glu^276^, Lys^279^ and Phe^280^. The summary of ligand interactions compared to the crystallographic reference is shown in Table 4. It is worth mentioning that similar results were found for the PTP1B_1–400_ structure (Table S3).

**Table 4:** Interactions found by molecular docking between PTP1B and computational hits.

1C83 (catalytic site)		Lys_120	Ser_216	Arg_221	Ala_217	Gly_220	Tyr_46	Val_49	Phe_182	Asp_48	Asp_181	Ser_222	Ala_217	Cys_215	Ile_219				
	OAI	X	X	X	X	X	X	X	X										
	**1a**	X	X	X	X	X	X	X	X	X	X				X				
	**2a**	X	X	X	X		X	X		X	X				X				
	**3a**	X	X		X	X	X	X		X	X				X				
	**4a**	X	X	X	X	X	X	X	X	X	X		X	X	X				
	**5a**	X	X	X	X		X	X	X	X	X			X	X				
	**6a**	X	X	X	X	X	X	X	X	X	X			X					
	**7a**	X	X		X	X	X	X	X	X	X	X							
	**8a**				X		X	X	X					X					

1T49 (allosteric site)		Asn_193	Glu_276	Phe_196	Asn_193	Leu_192	Lys_279	Phe_280	Ile_281	Gly_277	Ala_189	Pro_188	Lys_197	Ser_187	Arg_268	Leu_272	Phe_194	Glu_186	Leu_195
	892	X	X	X	X	X	X	X	X										
	**1b**		X	X	X	X	X	X	X	X	X	X							X
	**2b**			X	X	X	X	X	X	X	X	X		X	X	X		X	
	**3b**	X	X	X		X	X	X		X	X		X						
	**4b**		X	X	X	X	X	X		X	X	X	X	X			X		
	**5b**	X	X	X		X	X	X		X	X		X						
	**7a**	X	X			X		X		X	X	X		X					

**Scheme 1:**
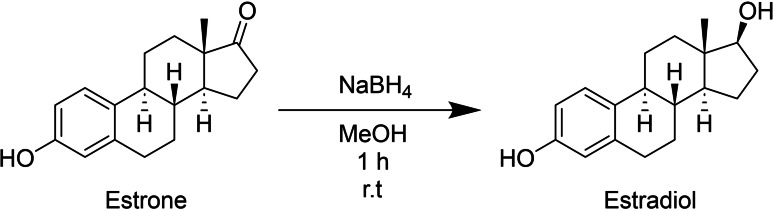
Synthesis of estradiol from estrone.

Seven compounds (**1a**–**6a** and **8a**) were purchased from Sigma Aldrich, and only estradiol (**7a**) was synthesized by reducing the 17‐carbonyl group of estrone with sodium borohydride (Scheme 1). The product obtained a 90 % yield with a melting point of 176–182 °C, which agrees with previous literature reports (178.5 °C). In addition, the compound was characterized using ^1^H NMR, which agrees with previous reports.^[38]^ See supplementary material (Figure S2).

### 2.4 *In Vitro* Assays

#### 2.4.1 *In Vitro* Evaluation of the Inhibitory Activity of the Compounds on PTP1B

**Figure 6:**
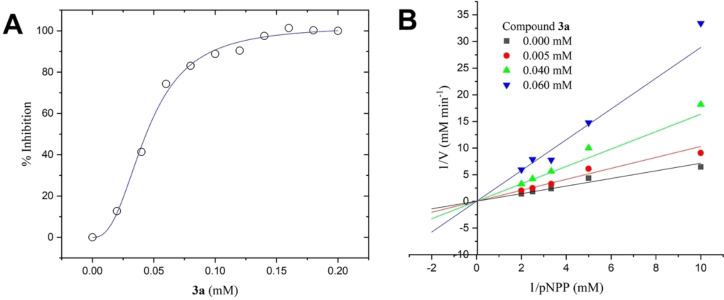
Evaluation of compound **3a** on PTP1B. (A) Dose‐response curve for determination of IC_50_; (B) Lineweaver‐Burk plot for determination of inhibition type.

Compounds **1a–8a**, resulting from the docking studies at the PTP1B's catalytic, were selected to determine its inhibitory activity against PTP1B by using a spectrophotometric assay.[^39^, ^40^] The compounds to be assessed included estradiol (**7a**), which was also found to target the allosteric site. Other estradiol derivatives, such as **3b**, **4b**, and **5b**, also showed good *in silico* binding energies on the allosteric site; however, since all of them are structural analogues, only the evaluation of estradiol was considered because it was the most representative. On the other hand, fenofibrate (**1b**) was also found to bind at the allosteric site. Fenofibrate is a prodrug of fenofibric acid **8a**, which also binds to the catalytic site. Thus, only **8a** was representatively included in the *in vitro* studies. The compounds were evaluated at a concentration of 2 mM, taking ursolic acid (UA)^[41]^ as a positive control, Figure S3. Only one compound showed significant inhibitory activity: 13‐*cis*‐retinoic acid (**3a**). Subsequently, the inhibitory concentration (IC_50_) for **3a** was determined to be 0.044±0.001 mM (Figure 6). The IC_50_ is eight times higher than UA (IC_50_=0.0056 mM).

**Figure 7:**
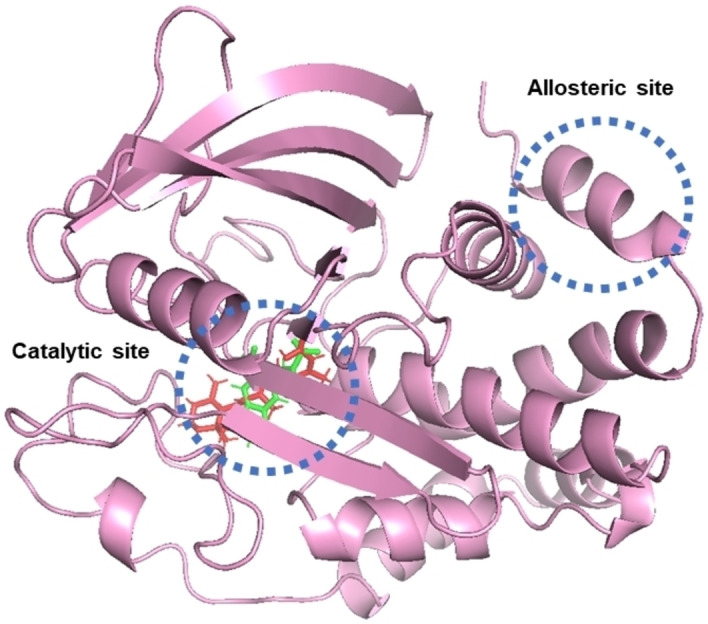
Representation of PTP1B binding sites. The binding mode of **3a** (red) and the crystallographic ligand OAI (green) obtained by Autodock 4.2 are shown at the catalytic site.

To determine the type of inhibition of compound **3a**, enzymatic tests were carried out.[^42^, ^43^] The results showed that **3a** is a competitive inhibitor according to the parallel lines observed in the Lineweaver‐Burk plot (Figure 6B).^[44]^ The kinetic parameters obtained were V_max_=36.06±1.47 mM/min, K_m_=25.71±1.06 mM/min, and K_i_=0.0234±0.002 mM. These results agree with the docking results, where it is estimated that **3a** binds to the catalytic site of the PTP1B enzyme, Figure 7.

Interestingly, **3a** (a vitamin A metabolite) has never been reported as PTP1B inhibitor.

During the 1960s, the Swiss‐based Roche Laboratories studied a compound named **3a** as a possible treatment for skin cancer. However, by 1971, it was ineffective as a cancer treatment. Fortunately, it was discovered that the compound could be used as an effective treatment for acne.^[45]^ It was marketed under the brand names Accutane® and Roaccutane®.

Compound **3a** has several properties that make it an effective acne treatment, including its ability to suppress sebum production, reduce the size of sebaceous glands, prevent follicles from clogging with small scales, and inhibit the growth of Propionibacterium acnes bacteria, which are often involved in acne pathology. Additionally, it has some anti‐inflammatory properties. It is important to note that DE_50_ for humans is 0.4 mg/kg.^[46]^

This compound is particularly indicated for severe acne and cases where other treatments such as antibiotics, ointments, creams, or new skin hygiene habits have failed. It is available in various forms, such as hydroalcoholic solutions, gels, suspensions, o/w and w/s emulsions, and ointments. These different formulations make it easier to check the possible therapeutic use of the compound as a PTP1B inhibitor.

It is worth mentioning that **3a** (also known as isotretinoin) has been identified as a teratogen. The ingestion of isotretinoin during pregnancy, or even shortly before conception, can lead to a high risk of congenital malformations affecting various organ systems. These defects can include craniofacial abnormalities, cardiovascular malformations, and central nervous system deformities.^[47]^ Due to its teratogenic effects, isotretinoin is contraindicated in pregnant women and women of childbearing potential who are not using effective contraception.^[48]^ Therefore, these toxicological implications should be considering for the potential therapeutic use of **3a** as a PTP1B inhibitor.

### 2.5 Molecular Dynamics Simulation

**Figure 8:**
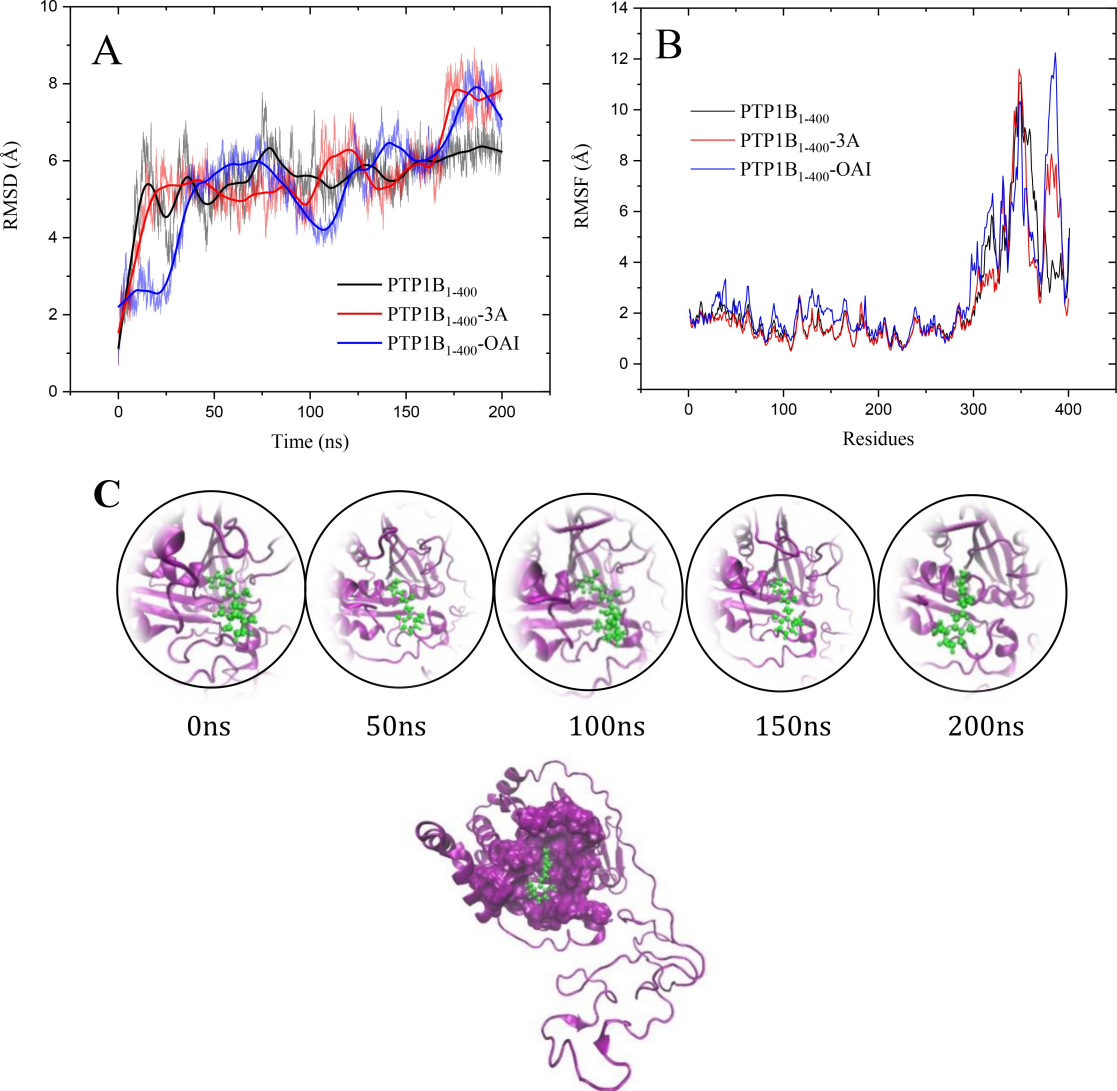
MDS analysis of PTP1B_1–400_ and the PTP1B_1–400_‐**3a** and PTP1B_1–400_‐OAI complexes. A) RMSD vs time, B) RMSF vs Residues and C) structural model of the PTP1B_1–400_‐**3a** complex and panels every 50 ns of MDS; the enzyme is shown in purple cartoons, the residues that participate along the MDS are shown in purple surface and the ligand **3a** is shown in green sticks.

Molecular dynamics simulation (MDS) studies indicated that the complexes remained bound to PTP1B_1–400_ throughout the analysis.^[49]^ The MDS of the PTP1B_1–400_ and the PTP1B_1–400_‐**3a** and PTP1B_1–400_‐OAI complexes were made for 200 ns, generally observing that the inhibitor **3a** remains throughout the entire trajectory in the catalytic site of the enzyme, which is in harmony with the results of enzyme kinetics and docking; while the OAI ligand seems to bind two regions of the enzyme in the first 60 ns the catalytic site and in the last 140 ns a bidentate site (Figure 8, Video 2 and 3), these bidentate sites are sites close to the catalytic site that can bind some small inhibitors.^[50]^ Regarding the RMSD of the complexes, these were very similar to those of the enzyme without ligands (5–6 Å). With respect to the fluctuation per residue (RMSF), only small differences are observed in the intrinsically unstructured region (300–400 amino acids).

**Figure 9:**
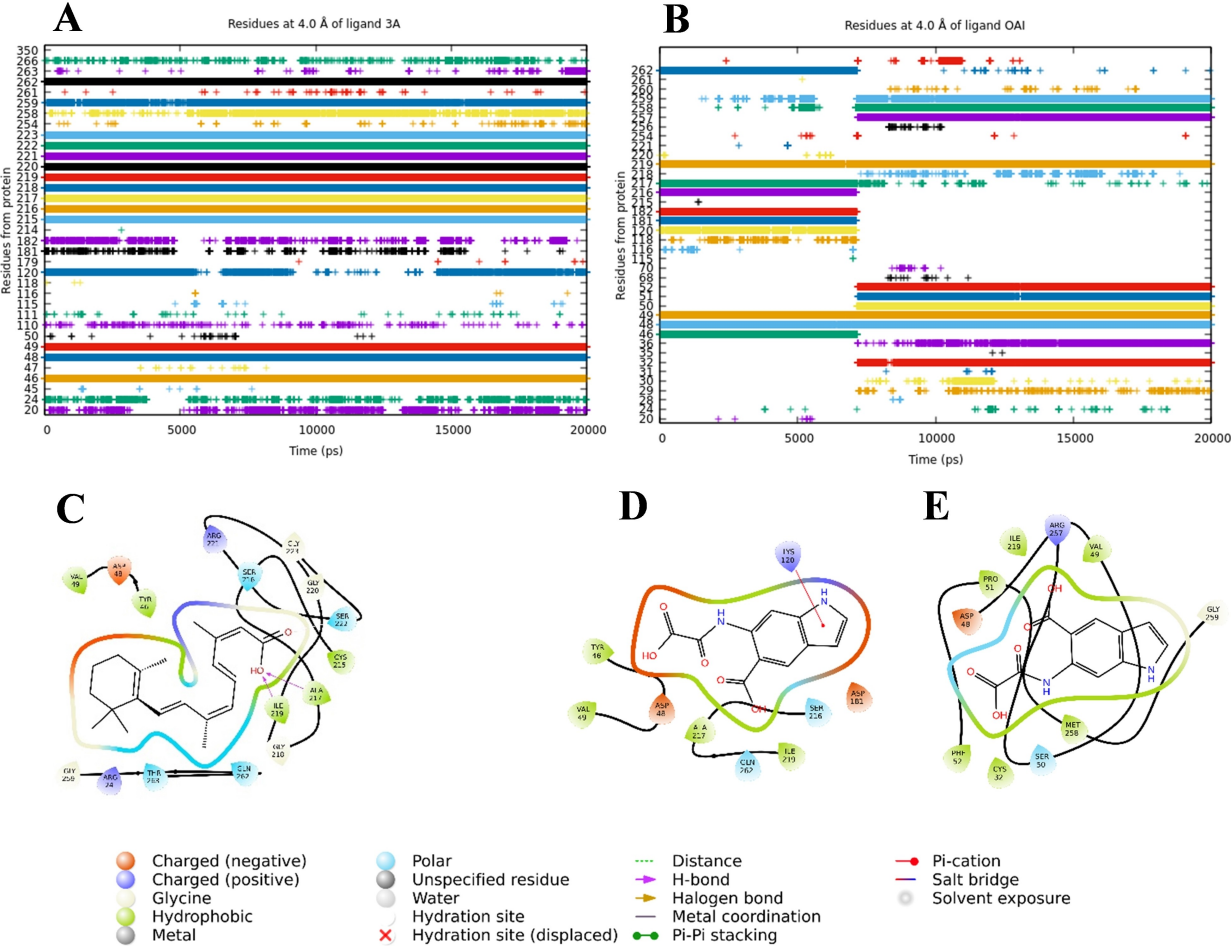
Residue analysis from the MDS in the protein‐ligand complex. Panel A and B, show the residues that interact with 4 Å of the ligands **3a** and OAI, respectively. 2D representation of the residues of the PTP1B_1–400_‐**3a** complex (C), PTP1B_1–400_‐OAI from 0 ns–60 ns (D) and PTP1B_1–400_‐OAI from 60 ns–200 ns (E). The graphics were made with the origin program and the 2D representations with the maestro program, Schrodinger Suite 2024–2.

We performed an analysis of residues with which inhibitors **3a** and OAI interact at 4 Å from these. Figure 9 and Table 5 show the results obtained where for the PTP1B_1–400_‐**3a** complex the residues correspond to those previously reported for the catalytic site.^[29]^ For the PTP1B_1–400_‐OAI complex, the analysis was carried out according to the two sites that it binds during the MDS, for the first (0–60 ns) it corresponds to the catalytic site and for the second (60–200 ns) it corresponds to a bidentate site of the enzyme, residues from the catalytic site are found in both areas. Additionally, we calculate the binding free energy of the complexes using the MM/PBSA methods, obtaining that the most stable complex corresponds to PTP1B_1–400_‐**3a**, since the calculated ΔG theoretical corresponds to −45.11±6.11 kcal/mol, while the PTP1B_1–400_‐OAI complex it was −16.61±1.99 and −20.74±5.12 kcal/mol to for the catalytic and bidentate site, respectively.

**Table 5:** Amino acid residues along the simulation trajectory of PTP1B_1–400_‐**3a** and PTP1B_1–400_‐OAI.

	Interaction residuals
PTP1B_1–400_‐**3a**	Try^46^, Asp^48^, Val^49^, Cys^215^, Ser^216^, Ala^217^, Gly^218^, Ile^219^, Gly^220^, Arg^221^, Ser^222^, Gly^223^, Met^258^, Gly^259^, and Gln^262^
PTP1B_1–400_‐OAI (0 ns at 60 ns)	Try^46^, Asp^48^, Val^49^, Gly^120^, Asp^181^, Phe^182^, Ser^216^, Ala^217^, Ile^219^, and Gln^262^
PTP1B_1–400_‐OAI (60 ns at 200 ns)	Cys^32^, Lys^36^, Aps^48^, Val^49^, Ser^50^, Pro^51^, Phe^52^, Ile^219^, Arg^257^, Met^258^, and Gly^259^

## 3 Materials and Methods

### 3.1 Computational Study

#### 3.1.1 Construction of the Molecular Docking Database

The compounds considered for this study were obtained from ChEMBL with code 335, https://www.ebi.ac.uk/chembl/ version 14 (accessed on 3 November 2021). A total of 4285 PTP1B inhibitors, 605 α‐glucosidase inhibitors, 2135 PPAR‐γ ligands and 10849 drugs approved or in different phases of research (pharmaceuticals) were obtained. Compounds with activity against PTP1B, α‐glucosidase and PPAR‐γ were filtered employing the DataWarrior program applying the following criteria^[23]^: 1) only compounds with molecular weight between 160–1000 g/mol were considered, 2) duplicate compounds were eliminated, 3) compounds with activity values greater than 100 μM were eliminated. For drugs, only exclusion criteria 1 and 2 were applied.

Subsequently, the databases were classified by chemotype at the Scaffold resolution level following the Murcko protocol implemented in the DataWarrior software.^[23]^ The classification consists of eliminating the side chains of each molecule; on the other hand, the order of the bonds, the types of atoms and the connectors between ring systems are preserved. Exocyclic double bonds and carbonyl, thiocarbonyl, imine, sulfone and sulfoxide groups were considered as part of the cyclic system when they are part of a ring or are on the connectors between rings, and an example is shown in Figure S1A.

A selection of compounds for virtual screening studies on PTP1B was carried out. This consisted of a chemotype comparison between molecules in the database of compounds active on PTP1B and test databases, including compounds with activity on α‐glucosidase and PPAR‐γ; in addition, this same comparison was carried out with a database of commercial drugs (Drugs), Figure 3.

#### 3.1.2 Virtual Screening by Molecular Docking on PTP1B

##### 3.1.2.1 Receptor and Ligand Preparation

The structures of PTP1B with the codes 1C83 (catalytic site) and 1T49 (allosteric site) were obtained from the Protein Data Bank. The protein was saved in *.pdb format and minimized using the YASARA Energy Minimization Server platform.^[26]^ Using the YASARA structure, water molecules within the proteins were removed, and then a cubic simulation cell with an extension of 5 Å was created around the cocrystallized ligands OAI (for PDB ID: 1C83) and 892 (for PDB ID: 1T49). The cocrystallized ligands were then removed, and the structures of both proteins were saved in *.sce format.

Conversely, all 1075 ligands, including α‐glucosidase inhibitors, PPAR‐γ ligands and approved drugs, were subjected to geometric optimization using the MMFF94 force field implemented in the DataWarrior 5.5.0 program. The ligands were then saved as an *.sdf file and submitted to YASARA structure.

##### 3.1.2.2 Molecular Docking Studies

Molecular docking studies were carried out using Vina and Autodock (implemented in YASARA structure) as follows:

Two previously saved *.sce files for either 1C83 or 1T49 were chosen as a macro target for Vina docking simulations. The *dock_screening.mcr* macro, previously modified to include 100 runs, was selected for the simulations. After analyzing the results, the best compounds were chosen and a new *.sdf file was created using Datawarrior 5.5.0. These compounds were then subjected to docking simulations with Autodock using the *dock_screening.mcr* macro, which had now been modified to run AutoDockLGA with 100 runs. For docking studies using GOLD, the structures of the minimized structures of PTP1B (1C83 and 1T49) were exported to the GOLD program (version 2020). Hydrogens were added within a sphere of a 10 Å radius using the following parameters: 100 runs of genetic algorithms and 125000 operations. The CHEMPLP fitness function was chosen for analysis and for molecular docking classification.

In all cases, the LigRMSD platform (https://ligrmsd.appsbio.utalca.cl/) (accessed August 09, 2022) was used for validation, which was performed by comparison of the RMSD value of the co‐crystallized ligand with the docking result using the different programs. Interaction analysis and figure preparation were performed with the PyMOL visualization tool (PyMOL Molecular Graphics System v1.7.4), while 2D interaction diagrams were produced with the BIOVIA Discovery Studio 2020 visualizer (Dassault Systèmes, San Diego, CA, USA).

### 3.2 Compounds

The compounds found as computational hits were screened to select those that were commercially available or had a commercial synthetic precursor. The commercial derivatives or synthetic precursors were obtained from Sigma‐Aldrich (Toluca MEX, Mexico). Seven compounds were acquired as final products: camostat, NCX 4016, 13‐*cis*‐retinoic acid, tolcapone, estrone, xenalipin and fenofibric acid. Estradiol was synthesized by reduction of estrone with sodium borohydride using the procedure described in the supplementary information.

### 3.3 Biological Assays

#### 3.3.1 *In Vitro* Evaluation of the Inhibitory Effect of the Compounds on PTP1B

The spectrophotometric method previously described was used.^[39]^ The compounds and the positive control were dissolved in dimethylsulfoxide (DMSO). Aliquots of 0–10 μL solution of the compounds to be tested (in triplicate) were incubated with 85 μL of the enzyme (66 nM) in Tris [50 mM; Tris‐HCI Buffer, pH 6.8] and 5 microliters of the substrate (pNPP, 10 mM), for 15 min at 37 °C. After incubation, the absorbance was determined. The inhibitory activity for the compounds was determined as a percentage compared to the blank (Tris) according to the following equation:

(1) $$\%PTP1B=\left(1-\frac{A_{415t}}{A_{415c}}\right)X\;100\;\%$$

Where %PTP1B is the percentage inhibition, A_415t_ is the corrected absorbance of the tested compound (A_415 final_‐A_415 initial_), and A_415c_ is the corrected absorbance of the blank (A_415 final_ blank‐A415 _initial target_). The IC_50_ was calculated by regression analysis using the following equation:

(2) $$\%Inhibition=\frac{A_{100}}{1+{\left(\frac{I}{{CI}_{50}}\right)}^{s}}$$

Where A_100_ is the maximum inhibition, I is the inhibitor concentration, CI_50_ is the concentration required for 50 % inhibition of enzyme activity, and s is the cooperative degree.

#### 3.3.2 Enzyme Kinetics Studies

The type of inhibition was determined by employing enzymatic kinetics on compound **3a** using the method previously described by Lorsch.^[42]^ The assay was carried out with different concentrations of inhibitor (0, 0.005, 0.040 and 0.060 mM) and substrate (0.10, 0.20, 0.30, 0.40, and 0.50 mM). Kinetic parameters (V_max_, K_m_, and K_i_) were obtained by nonlinear regression fit analysis using Origin 2018 software.

### 3.4 Molecular Dynamics Simulations

For the Molecular dynamics simulations (MDS), the proteinwas first validated with the pdb4amber script, part of AmberTools, the GAFF forcefield was chosen for ligands.^[51]^ Then, the coordinates and topologies of the complexes were prepared using the tLEAP module of AMBER23.[^52^, ^53^, ^54^] Subsequently, hydrogens and some missing atoms were added to the structure and the complex using the tLEAP module with force camp protein.ff19SB^[54]^; after that, an optimization of the hydrogen bond network was performed in order to increase the stability of the solute and to be able to have a pKa prediction to adjust the protonation states of the protein residues at pH 7.4. At this point, Na^+^ counterions were included to neutralize the system. The complexes were solvated in an octahedral box of water molecules using the TIP3P model; the box's boundaries were 12 Å from the protein surface.

MDS were performed at 1 atm and 315 K, maintained with the Berendsen barostat and thermostat. Periodic boundary conditions and Ewald particle mesh sums (1 Å spacing) were used to treat long‐range electrostatic interactions. To calculate direct interactions, a 10 Å cutoff was used. On the other hand, to satisfy the binding constraints, the SHAKE algorithm was used, thus allowing the use of a time step of 2 fs to integrate Newton's equations as mentioned in the Amber package. Amber force field parameters protein.ff19SB were also used for all residuals. The calculations were performed using a graphics processing unit‐accelerated MD engine in AMBER (pmemd.cuda), a program package running entirely on CUDA‐enabled GPUs.[^55^, ^56^] Simulations were performed on Ubuntu 22.04 Workstation with a Nvidia^®^ Gigabyte GeForce RTX 4090 GPU, yielding a max performance of 296 ns/day

This protocol begins with an initial structure minimization before pressure equilibration at 315 K and 1.0 atm, respectively. Before MDS begins to produce, the system is equilibrated with 500 ps. Each complex produced 200 ns of MDS. The CPPTRAJ tool by AMBER23 utilities was used to perform all analyses.[^53^, ^57^] RMSD calculations were performed considering C, CA, and N. Graphs were created using Origin 2018. VMD. Maestro Schrödinger, and PyMOL were used to visualize and create the MDS images.[^54^, ^58^]

Binding free energies calculated by molecular mechanics/poisson boltzmann surface area (MM/PBSA). This method involves a combination of molecular mechanic's energy with implicit solvation models to calculate binding free energies. In MM/PBSA,^[59]^ binding free energy (ΔG_bind_) between a ligand (L) and a target (T) to form a complex is calculated as:

(3) $${\Delta G}_{bind}=\;\Delta H-T\Delta S\approx {\Delta E}_{MM}+{\Delta G}_{Sol}-T\Delta S$$

(4) $${\Delta E}_{MM}={\Delta E}_{Internal}+{\Delta E}_{Electrostatic}+{\Delta E}_{Vdw}$$

(5) $${\Delta G}_{Sol}={\Delta G}_{PB}+{\Delta G}_{SA}$$

where Δ*E*_MM_, Δ*G*_Sol_ and −*T*Δ*S* are the changes of the gas phase molecular mechanics energy, the solvation free energy and the conformational entropy upon binding, respectively. Δ*E*_MM_ comprises Δ*E*_Internal_ (bond, angle and dihedral energies), Δ*E*_Electrostatic_ (electrostatic energies), and Δ*E*_Vdw_ (van der Waals energies). Δ*G*_Solv_ is the sum of electrostatic solvation energy (polar contribution) ‐Δ*G*_PB_‐ and non‐electrostatic solvation component (non‐polar contribution) ‐Δ*G*_SA_‐. The polar contribution is calculated using the Poisson‐Boltzmann surface area model, while the non‐polar energy is estimated from the solvent accessible surface area (*SASA*). The conformational entropy change (−*TΔS*) was computed by normal mode analysis from a set of conformational snapshots taken from the MD simulations.[^60^, ^61^]

## 4 Conclusions

This study aimed to identify potential drugs that could act as prototype drugs for the treatment of T2D by inhibiting PTP1B. To achieve this, a database was built with drugs on the market, which is an excellent strategy for drug repositioning, followed by a computational screening, using three programs: Autodock Vina, Autodock 4.2 and GOLD (showing better results with Autodock 4.2, known for its Lamarckian Genetic Algorithm (LGA) search algorithm and its ability to model the flexibility of target proteins), based on a drug repositioning approach using chemoinformatics, molecular docking, MDS and biological assays on PTP1B. Our theoretical and experimental studies identified **3a** as a competitive inhibitor of PTP1B.

The compound is interesting because it is a metabolite of vitamin A with anti‐inflammatory properties and is commonly used to treat acne. As a result, it has known safety and properties and is already commercially available. Therefore, this compound holds promise for future studies as a possible treatment for T2D. In addition, docking and MDS suggest that 13‐*cis*‐retinoic acid (**3a**) binds to the catalytic site with better stability than that of the co‐crystallized ligand (OAI), which complements the experimental results, therefore it is very promising to be considered a good PTP1B inhibitor. To conclude, **3a** is the first time that it has been described as an inhibitor of PTP1B and, therefore, with possible antidiabetic properties.

## Conflict of Interests

The authors declare no conflict of interest.

5

## Supporting information

As a service to our authors and readers, this journal provides supporting information supplied by the authors. Such materials are peer reviewed and may be re‐organized for online delivery, but are not copy‐edited or typeset. Technical support issues arising from supporting information (other than missing files) should be addressed to the authors.

Supporting Information

Supporting Information

Supporting Information

Supporting Information

## Data Availability

The data that support the findings of this study are available in the supplementary material of this article.
