# Supplementary material for: Virtual and in Vitro Screening Employing a Repurposing Approach Reveal 13‐cis‐Retinoic Acid is a PTP1B Inhibitor
Source: ChemMedChem. 2024 Oct 8;19(23):e202400452. doi: 10.1002/cmdc.202400452 (PMC11617665; doi:10.1002/cmdc.202400452)
Supplement: Supplementary file 1 — Supporting Information [file CMDC-19-e202400452-s003.pdf]

ChemMedChem

Supporting Information

Virtual and in Vitro Screening Employing a Repurposing Approach Reveal 13-*cis*-Retinoic Acid is a PTP1B Inhibitor

Reyna Del Carmen Navarrete-Mondragón, Francisco Cortés-Benítez, Jessica Elena Mendieta-Wejebe, Martin González-Andrade,* and Jaime Pérez-Villanueva*

Supporting information

Virtual and in vitro Screening Employing a Repurposing Approach Reveal 13-Cis-retinoic acid is a PTP1B Inhibitor

Reyna Del Carmen Navarrete-Mondragón[1], Francisco Cortés-Benítez[2], Jessica Elena Mendieta-Wejebe[3], Martin González-Andrade[4]*, Jaime Pérez-Villanueva[2]*

1 Doctorado en Ciencias Biológicas y de la Salud, Universidad Autónoma Metropolitana (1), Ciudad de México 04960, México; reynarunner3@gmail.com

2 Departamento de Sistemas Biológicos, División de Ciencias Biológicas y de la Salud, Universidad Autónoma Metropolitana–Xochimilco (1), Ciudad de México 04960, México; jpvillanueva@correo.xoc.uam.mx (J.P.-V.); jcortesb@correo.xoc.uam.mx (F.C.-B.)

3 Laboratorio de Biofísica y Biocatálisis, Sección de Estudios de Posgrado e Investigación, Escuela Superior de Medicina, Instituto Politécnico Nacional, Plan de San Luis y Salvador Díaz Mirón s/n, Casco de Santo Tomás, Miguel Hidalgo, Ciudad de México 11340, México; jesmenwej@yahoo.com (J.E.M.-W.)

4 Departamento de Bioquímica, Facultad de Medicina, Universidad Nacional Autónoma de México, Ciudad de México 04510, México; martin@bq.unam.mx

*****Correspondence:

(J.P.-V.) jpvillanueva@correo.xoc.uam.mx; Tel.: +52-5-54-83-72-59; Fax: +52-5-55-94-79-29

(M.G.-A.) martin@bq.unam.mx; Tel.: +52 (55) 56232254

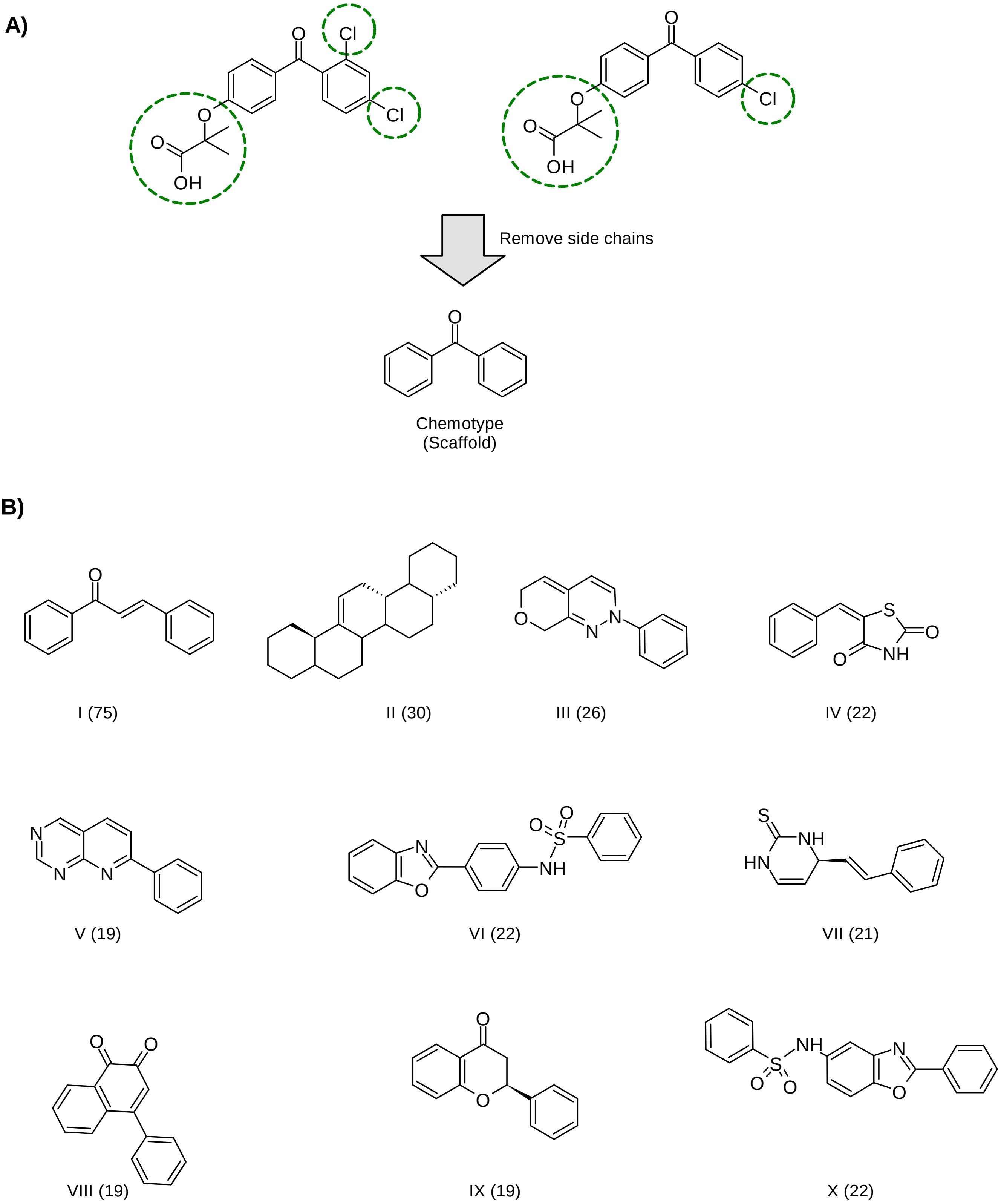

Figure S1. Chemotype classification of molecular databases. A) Example of Chemotype classification using the scaffold resolution level. B) Examples of the most frequent chemotypes found in the PTP1B database.

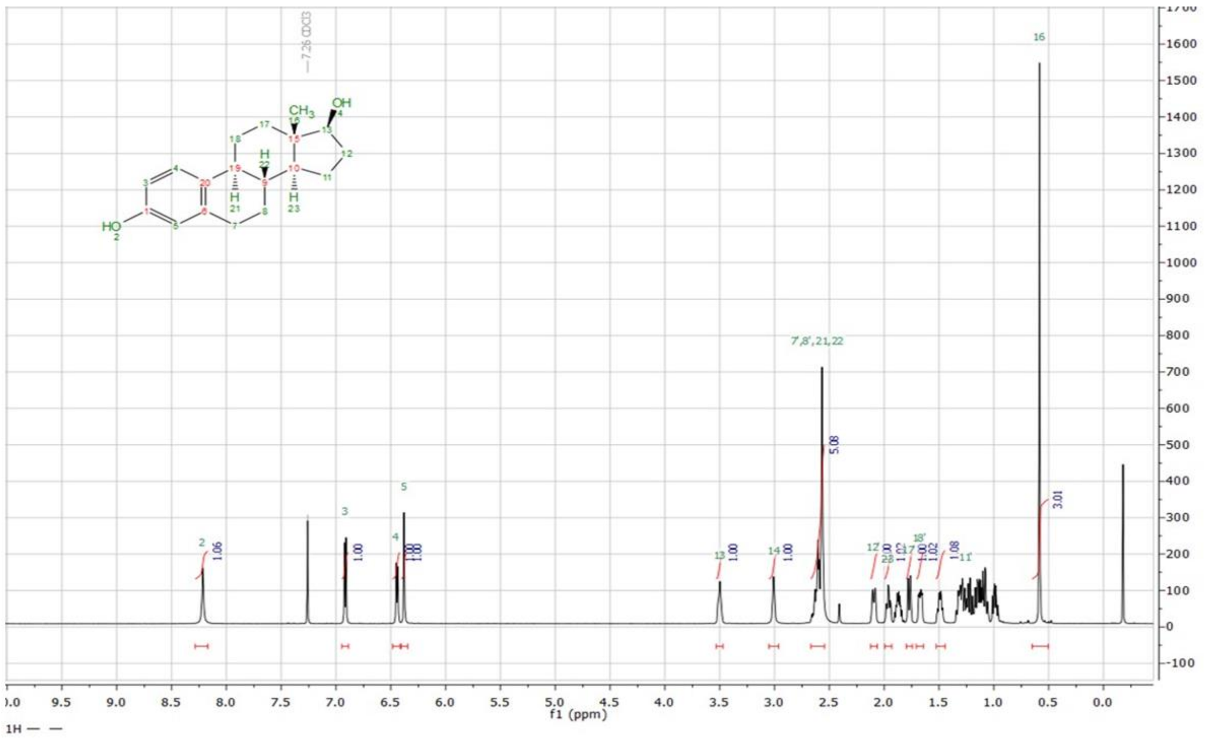

Figure S2. 1H NMR (600 MHz, $CDCl_3$) for estradiol.

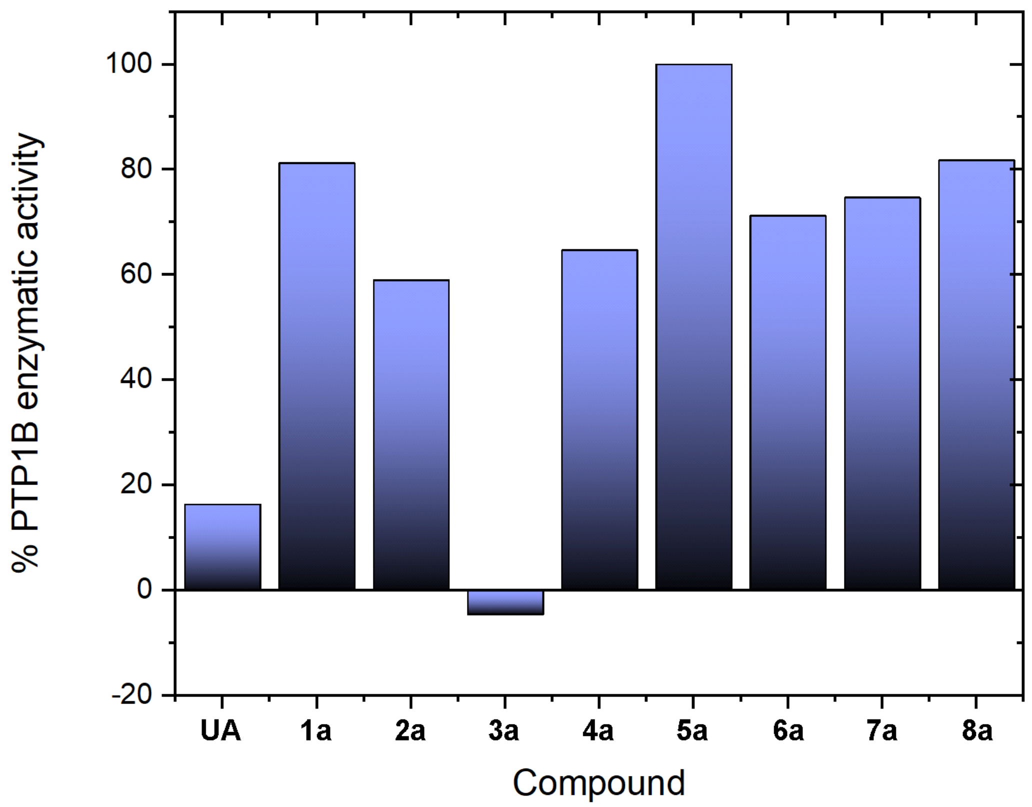

Figure S3. In vitro screening of selected compounds against PTP1B.

Table S1. Top 50 computational consensus hits on PTP1B (1C83) employing three docking programs

Smiles Code	Autodock Vina	Autodock 4.2	Gold	1-Z_{Vina}	1-$Z_{AD4.2}$	Z_{Gold}
Nc(c1ccccc1c(S(O)(=O)=O)c1)c1/N=N/c(cc1)ccc1-c(cc1)ccc1/N=N/c(cc(c1ccccc11)S(O)(=O)=O)c1N	-10.04	-10.04	39.90	1	0.909	0.804
Cc(cc(cc1)-c(cc2)cc(C)c2/N=N/c2ccc(c(S(O)(=O)=O)cc(S(O)(=O)=O)c3N)c3c2O)c1/N=N/c(ccc(c1c(c(S(O)(=O)=O)c2)N)c2S(O)(=O)=O)c1O	-9.92	-7.44	26.04	0.971	0.474	0.523
C[C@@H](C(O)=O)c(cc1)cc(F)c1-c1ccccc1	-9.16	-8.96	39.49	0.79	0.729	0.796
C[C@@](COc1ccc(C[C@H](C(N2)=O)SC2=O)cc1)(CC1)Oc(c(C)c2C)c1c(C)c2O	-9.07	-10.58	43.07	0.773	1	0.869
CC(C(O)=O)c(cc1)ccc1-c1cccc(F)c1	-9.06	-9.00	35.30	0.771	0.735	0.711
C[C@@H]([C@@H]([C@H]([C@H]1O)O)O)O[C@H]1OC[C@H]([C@H]([C@@H]([C@H]1O)O)O)O[C@H]1Oc1cc(O[C@@H](CC2=O)c(cc3)cc(O)c3OC)c2c(O)c1	-8.98	-9.36	28.57	0.751	0.795	0.574
**CN(C)C(COC(Cc(cc1)ccc1OC(c(cc1)ccc1NC(N)=N)=O)=O)=O**	**-8.97**	**-9.95**	**39.89**	**0.749**	**0.894**	**0.804**
C[C@](CC1)([C@@H](CC2)[C@H](CC3)[C@H]1c(cc1)c3cc1O)[C@@]2(C#CC)O	-8.86	-9.26	31.92	0.724	0.779	0.642
C[C@](CC1)([C@@H](CC2)[C@H](CC3)[C@H]1c(cc1)c3cc1O)[C@@]2(C#C)O	-8.71	-9.22	31.20	0.690	0.772	0.627
OC(COc(cc1)ccc1C(/C=C/c(cc1)ccc1C(O)=O)=O)=O	-8.68	-9.80	34.46	0.681	0.869	0.694
**C[C@](CC1)([C@@H](CC2)[C@H](CC3)[C@H]1c(cc1)c3cc1O)[C@@H]2O**	**-8.62**	**-8.82**	**32.49**	**0.668**	**0.705**	**0.654**
C[C@](CCC=C(C)C)([C@@H](Cc1c2Oc(cc3)c4cc3O)Oc1c(CC=C(C)C)c(O)c2C4=O)O	-8.61	-9.36	37.96	0.666	0.795	0.765
COc1ccc(cc(-c(c(CC2)c3)cc4c3OCO4)[n+]2c2)c2c1OC	-8.57	-8.86	28.65	0.657	0.712	0.576
NS(c(cc(cc1)C(c(c(Cl)c2Cl)ccc2OCC(O)=O)=O)c1Cl)(=O)=O	-8.56	-10.35	35.68	0.653	0.961	0.719
Cc(cc(cc1)-c(cc2)cc(C)c2/N=N/c(c(S(O)(=O)=O)cc2cc(S(O)(=O)=O)cc(N)c22)c2O)c1/N=N/c(c(S(O)(=O)=O)cc1cc(S(O)(=O)=O)cc(N)c11)c1O	-8.54	-8.74	38.71	0.650	0.692	0.780
CC(C)=CCC/C(/C)=C/CC/C(/C)=C/Cc(c1c(cc2O)Oc(cc(cc3O)O)c3C1=O)c2O	-8.50	-9.09	46.45	0.641	0.750	0.937
Oc(c(S(O)(=O)=O)cc1cc(S(O)(=O)=O)ccc11)c1/N=N/c(c1ccccc11)ccc1S(O)(=O)=O	-8.47	-9.18	43.94	0.634	0.765	0.886
CC(Cc1cccc(C(F)(F)F)c1)NC(COc(cc1)ccc1NC(C)=O)=O	-8.42	-8.1	43.87	0.622	0.585	0.885
C[C@](CC1)([C@@H](C[C@H]2F)[C@H](CC3)[C@H]1c(cc1)c3cc1O)[C@H]2O	-8.37	-8.94	29.13	0.611	0.725	0.585
O=CC(c(cc1)ccc1-c(cc1)ccc1C(C=O)=O)=O	-8.33	-9.42	30.32	0.600	0.806	0.609
NC(c(cc1)cc(Br)c1OCCCOc(ccc(C(N)=N)c1)c1Br)=N	-8.29	-8.49	40.45	0.593	0.650	0.816
**CC(Oc(cccc1)c1C(Oc1cccc(CO[N+]([O-])=O)c1)=O)=O**	**-8.28**	**-10.52**	**35.82**	**0.590**	**0.989**	**0.721**
COc(ccc(C(Oc1c2c(OC)cc(OCC(O)=O)c1)=CC2=O)c1)c1OC	-8.25	-9.56	0.306	0.582	0.829	0
C[C@](CC1)([C@@H]([C@H]([C@H]2O)O)[C@H](CC3)[C@H]1c(cc1)c3cc1O)[C@H]2O	-8.23	-8.31	33.95	0.577	0.620	0.683
**C[C@](CC1)([C@@H](C[C@@H]2O)[C@H](CC3)[C@H]1c(cc1)c3cc1O)[C@H]2O**	**-8.22**	**-9.24**	**27.26**	**0.575**	**0.775**	**0.547**
CCCCCCc1cc(S(C)(=N)=O)cc2c1Oc(ccc(C(O)=O)c1)c1C2=O	-8.21	-9.58	42.06	0.574	0.832	0.848
CC(C)=CCC/C(/C)=C/COc(cc1Oc(cc2)c3cc2O)c(CC=C(C)C)c(O)c1C3=O	-8.20	-8.81	40.08	0.572	0.704	0.808
CCC(C(O)=O)c(cc1)ccc1-c1ccccc1	-8.19	-8.96	34.15	0.568	0.729	0.687
CCOC(C[C@@H](C#C)NC(CCC(Nc(cc1)ccc1C(N)=N)=O)=O)=O	-8.19	-7.16	41.94	0.567	0.428	0.846
**Cc(cc1)ccc1C(c(cc1O)cc([N+]([O-])=O)c1O)=O**	**-8.14**	**-9.63**	**30.38**	**0.557**	**0.841**	**0.611**
OC(CCN(C(/C(/S1)=C/c2cc(OCc(cc3)ccc3F)ccc2)=O)C1=S)=O	-8.14	-10.53	37.28	0.557	0.991	0.751

NC(c(cc1)ccc1OCCCOc(cc1)ccc1C(N)=N)=N	-8.10	-7.27	27.13	0.547	0.446	0.545
Cc(cc(cc1)C(C2)=NOC2(C(F)(F)F)c2cc(Cl)cc(Cl)c2)c1C(NCC(NCC(F)(F)F)=O)=O	-8.08	-9.21	39.97	0.543	0.770	0.806
NC(c1ccc(/C=C/c(cc2)ccc2C(N)=N)cc1)=N	-8.05	-7.25	27.21	0.536	0.443	0.546
CC(C)Oc(cccc1)c1C(Oc(cc1)c2cc1C(O)=O)=CC2=O	-8.05	-9.85	29.32	0.536	0.926	0.589
COc1ccc(C(CCC(O)=O)=O)c2ccccc12	-8.03	-9.69	37.34	0.531	0.851	0.752
CS(c(cc1)cc2c1Oc(ccc(C(O)=O)c1)c1C2=O)=O	-8.02	-9.52	32.46	0.529	0.822	0.653
NC(c1ccc(/C=C/c(ccc(C(N)=N)c2)c2O)cc1)=N	-8.00	-7.28	28.34	0.525	0.448	0.569
CC(C)=CCC/C(/C)=C/Cc(c(O)cc(/C=C/c1cccc(C(F)(F)F)c1)c1)c1O	-8.00	-7.89	34.38	0.523	0.550	0.692
Cc(cc1)cc(Cl)c1OCCCOc1ccc(CCC(O)=O)cc1	-7.98	-9.75	34.69	0.519	0.861	0.698
Oc(cc1O)cc(OC(c(cc2)cc(OP(O)(O)=O)c2O)=C2O)c1C2=O	-7.97	-9.22	35.58	0.518	0.772	0.716
CC(C(O)=O)Oc1cccc(C(c(cc2)ccc2Cl)=O)c1C	-7.97	-9.27	38.12	0.516	0.780	0.768
**OC(c(cccc1)c1-c1ccc(C(F)(F)F)cc1)=O**	**-7.96**	**-8.81**	**25.76**	**0.516**	**0.704**	**0.517**
CC(C)Cc1ccc([C@@H](C)C(O)=O)cc1	-7.94	-7.46	35.28	0.511	0.478	0.710
CC(C)(C)c(cc1)ccc1OCC(COc(cc1)ccc1C(O)=O)O	-7.94	-8.43	32.48	0.511	0.640	0.653
CC(/C=C(\C)/c(cc1)ccc1-c1ccccc1)=O	-7.93	-8.17	0.306	0.508	0.596	0
CC(C)=CCC/C(/C)=C/Cc(c(Oc(cc1)c2cc1O)c(c(O)c1CC(C(C)=C)O)C2=O)c1O	-7.92	-9.46	43.87	0.505	0.812	0.885
CC(C)=CCC/C(/C)=C/Cc(c(Oc(cc1)c2cc1O)c(c(O)c1CC=C(C)C)C2=O)c1O	-7.90	-8.61	40.55	0.501	0.670	0.817
CCC(O[C@@H](CC1)[C@@](C)(CC2)[C@@H]1[C@H](CC1)[C@H]2c(cc2)c1cc2OC(CC)=O)=O	-7.89	-9.4	30.88	0.499	0.802	0.621
CC1(C)C(/C=C/C(/C)=C/C=C/C(/C)=C/C(O)=O)=C(C)CCC1	-7.88	-7.87	17.30	0.497	0.546	0.345
CC(CC/C=C(\C)/CCC=C(C)C)(CCc(c1c(cc2O)Oc(cc(cc3O)O)c3C1=O)c2O)O	-7.88	-9.44	31.82	0.496	0.809	0.640
C[C@H](/C=C(\C)/C=C/C(NO)=O)C(c(cc1)ccc1N(C)C)=O	-7.88	-7.27	27.53	0.496	0.446	0.553
CC(C)S(NC[C@H](C)c(cc1)ccc1-c1ccc(CCNS(C)(=O)=O)cc1)(=O)=O	-7.86	-8.14	47.66	0.491	0.591	0.962
**CC1(C)C(/C=C/C(/C)=C/C=C/C(/C)=C\C(O)=O)=C(C)CCC1**	**-7.86**	**-8.06**	**25.90**	**0.491**	**0.578**	**0.520**
CC(C)Oc(cc1)cc2c1Oc(ccc(C(O)=O)c1)c1C2=O	-7.86	-9.07	35.97	0.491	0.747	0.724
**CC(C)(C(O)=O)Oc(cc1)ccc1C(c(cc1)ccc1Cl)=O**	**-7.86**	**-8.85**	**35.78**	**0.491**	**0.710**	**0.721**
C[C@H](C(SC[C@@H](C(O)=O)NC(C)=O)=O)c(ccc1c2)cc1ccc2OC	-7.85	-8.33	40.13	0.490	0.623	0.809
CC(C)NC[C@@H](COc1cccc(C2)c1OC[C@H]2O[N+]([O-])=O)O	-7.85	-10.14	34.73	0.490	0.926	0.699
Oc(cc(c1c2O)OC(c3ccccc3)=CC1=O)c2O	-7.84	-8.72	28.44	0.486	0.688	0.571
CC(C)(C(O)=O)Oc(cc1)ccc1C(c(cc1)ccc1Cl)=O	-7.83	-8.93	34.91	0.486	0.724	0.703

*Selected compounds

Table S2. Top 50 computational consensus hits on PTP1B (1T49) employing three docking programs

Smiles Code	Autodock Vina	Autodock 4.2	Gold	1-Z_{Vina}	1-$Z_{AD4.2}$	Z_{Gold}
Cc(cc(cc1)-c(cc2)cc(C)c2NNc(c(S([O-])(=O)=O)cc2cc(S([O-])(=O)=O)cc(N)c22)c2O)c1NNc(c(S([O-])(=O)=O)cc1cc(S([O-])(=O)=O)cc(N)c11)c1O	-13.87	-10.87	45.93	1	0.847	0.823
Cc(cc(cc1)-c(cc2)cc(C)c2NNc2ccc(c(S([O-])(=O)=O)cc(S([O-])(=O)=O)c3N)c3c2O)c1NNc(ccc(c1c(c(S([O-])(=O)=O)c2)N)c2S([O-])(=O)=O)c1O	-11.59	-10.96	47.35	0.777	0.861	0.847
**Nc(c1ccccc1c(S([O-])(=O)=O)c1)c1NNc(cc1)ccc1-c(cc1)ccc1NNc(cc(c1ccccc11)S([O-])(=O)=O)c1N**	**-11.32**	**-11.28**	**56.45**	**0.738**	**0.911**	**1**
Cc(c1c(-c(cc2C)cc(C)c2O[C@H](Cc2ccccc2)C([O-])=O)c2ccccc22)c(C)sc1c2Br	-10.63	-10.25	34.39	0.634	0.750	0.630
C[C@@](COc1ccc(C[C@H](C(N2)=O)SC2=O)cc1)(CC1)Oc(c(C)c2C)c1c(C)c2O	-9.36	-9.24	42.31	0.447	0.592	0.763
CC(C)=CCC/C(/C)=C/Cc(c(Oc(cc1)c2cc1O)c(c(O)c1CC=C(C)C)C2=O)c1[O-]	-9.36	-8.63	45.76	0.447	0.497	0.820
CCOC(/C=C(\C)/C=C/C=C(\C)/C=C/c(c(C)c1)c(C)c(C)c1OC)=O	-9.26	-7.67	-3.21	0.432	0.3478	0
C/C(/C=C/c(c(C)c1)c(C)c(C)c1OC)=C\C=C\C(\C)=C\C([O-])=O	-9.23	-7.79	23.75	0.426	0.366	0.451
[O-]S(c(cc1cc2S([O-])(=O)=O)ccc1c(NNc(c1ccccc11)ccc1S([O-])(=O)=O)c2O)(=O)=O	-9.22	-8.98	46.24	0.425	0.5522	0.828
Cc(cc(cc1)C(C2)=NO[C@@]2(C(F)(F)F)c2cc(Cl)cc(Cl)c2)c1C(NCC(NCC(F)(F)F)=O)=O	-9.17	-9.68	37.20	0.418	0.661	0.677
CC(C)=CCC/C(/C)=C/CC/C(/C)=C/Cc(c1c(cc2O)Oc(cc(cc3O)O)c3C1=O)c2O	-9.07	-8.68	34.35	0.404	0.505	0.629
CC(C)=CCCC([C@H](Cc(c(Oc(cc1)c2cc1O)c(c(O)c1CC=C(C)C)C2=O)c1O)O)=C	-8.97	-8.32	35.89	0.389	0.449	0.655
CCNC(/C=C(\C)/C=C/C=C(\C)/C=C/c(c(C)c1)c(C)c(C)c1OC)=O	-8.86	-7.83	24.58	0.373	0.372	0.465
CC(C)=CCC/C(/C)=C/Cc(c(O)cc(/C=C/c1cccc(C(F)(F)F)c1)c1)c1O	-8.86	-8.8	36.64	0.371	0.524	0.668
C[NH+](C)CCCNC(/C(/COc1cccc2ccccc12)=C/CCCCC(NO)=O)=O	-8.86	-8.92	33.08	0.371	0.542	0.608
CC(C)=CCc(c(O)c(CC=C(C)C)c(Oc(cc1)c2cc1O)c1C2=O)c1O	-8.84	-7.79	37.12	0.369	0.366	0.676
CN(C)C(COC(Cc(cc1)ccc1OC(c(cc1)ccc1[NH+]=C(N)N)=O)=O)=O	-8.82	-9.36	6.69	0.366	0.611	0.166
CC(C)(C)c(cc1)ccc1OC([C@H]1CC[C@H](C[NH+]=C(N)N)CC1)=O	-8.82	-8.91	35.87	0.366	0.541	0.655
Oc(cccc1C(c2cccc(O)c22)=O)c1C2=O	-8.76	-7.83	32.99	0.357	0.372	0.606
Cc1cc(/C=C/c(cc2)ccc2F)cc(C)c1O	-8.76	-7.31	-3.21	0.357	0.291	0
CC(C)=CCC/C(/C)=C/CC/C(/C)=C/CC/C(/C)=C/CC/C(/C)=C/CC/C(/C)=C/CC/C(/C)=C/CC(C(c1c2cccc1)=O)=C(C)C2=O	-8.76	-7.88	-3.21	0.356	0.380	0
CC(C)=CCC/C(/C)=C/Cc(c(O)cc(/C=C/c(cc1)ccc1C#N)c1)c1O	-8.75	-8.87	30.79	0.356	0.535	0.569
C[C@](CC/C=C(\C)/CC/C=C(\C)/CCC=C(C)C)(CC1)Oc(cc2)c1cc2O	-8.75	-8.08	31.06	0.356	0.411	0.574
CC(C)=CCC/C(/C)=C/Cc(c(Oc(cc1)c2cc1O)c(c(O)c1C[C@@H](C(C)=C)O)C2=O)c1O	-8.72	-8.12	34.82	0.351	0.418	0.637
CC(C)=CCC/C(/C)=C/Cc(c1c(cc2O)Oc3cc(O)c(CC=C(C)C)c(O)c3C1=O)c2OC	-8.70	-7.69	45.64	0.349	0.351	0.818
C=C(CC(c(cc1)ccc1-c(cccc1)c1Cl)=O)C([O-])=O	-8.70	-8.33	34.19	0.348	0.450	0.626
CC(C(C(C)=C1CCCCC#CCCCC#CCO)=O)=C(C)C1=O	-8.67	-8.5	35.91	0.344	0.477	0.655
C[C@](CC1)([C@@H](C[C@H]2F)[C@H](CC3)[C@H]1c(cc1)c3cc1O)[C@H]2O	-8.67	-8.25	26.22	0.344	0.438	0.493
CC[C@@](C)(Cc(cc1)ccc1OCCCOc(ccc(C(F)(F)F)c1)c1Cl)C([O-])=O	-8.67	-8.67	37.82	0.343	0.503	0.687
NC(Oc1ccc(Cc2ccccc2)cc1)=O	-8.66	-6.99	33.07	0.342	0.241	0.608
C[C@@H](C([O-])=O)Oc1cccc(C(c2ccccc2)=O)c1	-8.65	-7.36	31.48	0.340	0.299	0.581
CC(C)=CCC/C(/C)=C/Cc(c(O)cc(/C=C/c(cc1)ccc1Cl)c1)c1O	-8.65	-8.81	39.58	0.340	0.525	0.717
Cc(cc1O)c(Cc(c(C)c2)cc(S([O-])(=O)=O)c2O)cc1S([O-])(=O)=O	-8.63	-6.82	34.79	0.338	0.215	0.636

CC(C)=CCc(c(OC)c1)cc2c1OC(c(cc1)ccc1O)=CC2=O	-8.63	-8.73	38.96	0.338	0.513	0.706
COc1ccc(cc(-c(c(CC2)c3)cc4c3OCO4)[n+]2c2)c2c1OC	-8.63	-8.84	32.71	0.337	0.530	0.602
CC(C)=CCC/C(/C)=C/Cc(c(O)cc(/C=C/c(cc1)ccc1F)c1)c1O	-8.62	-8.74	5.28	0.336	0.514	0.142
CCC(O[C@@H](CC1)[C@@](C)(CC2)[C@@H]1[C@H](CC1)[C@H]2c(cc2)c1cc2OC(CC)=O)=O	-8.6	-8.54	27.56	0.333	0.483	0.515
Cc(cc1)c(C)c(Oc2c3cccc2CC([O-])=O)c1C3=O	-8.57	-7.35	35.01	0.329	0.297	0.640
C[C@](CC/C=C(\C)/CC/C=C(\C)/CCC=C(C)C)(CCc(c1c(cc2O)Oc(cc(cc3O)O)c3C1=O)c2O)O	-8.56	-8.07	33.09	0.328	0.410	0.608
CC(C)=CCC/C(/C)=C/Cc(c(O)cc(/C=C/c1ccc(C(F)(F)F)cc1)c1)c1O	-8.54	-8.43	39.02	0.324	0.466	0.707
CC(C)=CCc(c(OC)c1)cc(C(C2)=O)c1O[C@@H]2c(cc1)ccc1O	-8.53	-8.61	32.20	0.323	0.494	0.593
**C[C@](CC1)([C@@H](CC2)[C@H](CC3)[C@H]1c(cc1)c3cc1O)[C@@H]2O**	**-8.53**	**-8.23**	**25.31**	**0.322**	**0.435**	**0.478**
CC(C)c1cc(Cl)c(C)c(Cc(c(C)c(cc2C(C)C)Cl)c2O)c1O	-8.51	-7.22	29.14	0.320	0.277	0.542
CC(/C=C(\C)/c(cc1)ccc1-c1ccccc1)=O	-8.51	-7.19	-3.21	0.320	0.273	0
CC(C)=CCC/C(/C)=C/Cc(c(O)cc(/C=C/c1cc(F)cc(F)c1)c1)c1O	-8.50	-9.11	36.81	0.319	0.572	0.670
C[C@@H](Cc1cccc(C(F)(F)F)c1)NC(COc(cc1)ccc1NC(C)=O)=O	-8.50	-8.31	39.62	0.319	0.447	0.718
CC(C)=CCC/C(/C)=C/Cc(c(O)cc(/C=C/c1ccccc1)c1)c1O	-8.49	-8.73	33.84	0.318	0.513	0.621
**C[C@](CC1)([C@@H](CC2)[C@H](CC3)[C@H]1c(cc1)c3cc1OC)[C@@]2(C#C)O**	**-8.49**	**-8.17**	**28.05**	**0.317**	**0.425**	**0.524**
**CC(C)OC(C(C)(C)Oc(cc1)ccc1C(c(cc1)ccc1Cl)=O)=O**	**-8.48**	**-7.68**	**27.94**	**0.316**	**0.349**	**0.522**
NC(c1ccc(/C=C/c(cc2)ccc2C(N)=[NH2+])cc1)=[NH2+]	-8.47	-9.19	32.18	0.314	0.585	0.593
**C[C@](CC1)([C@@H](CC2)[C@H](CC3)[C@H]1c(cc1)c3cc1O)[C@@]2(C#C)O**	**-8.46**	**-8.17**	**24.41**	**0.313**	**0.425**	**0.463**
CC(C)=CCC/C(/C)=C/Cc(c(O)cc(/C=C/c(cc1)ccc1O)c1)c1O	-8.46	-9.21	30.09	0.313	0.588	0.558
C[C@](CC1)([C@@H](CC2)[C@H](CC3)[C@H]1c(cc1)c3cc1OC(N(CCCl)CCCl)=O)[C@H]2O	-8.46	-9.61	28.21	0.312	0.650	0.526
O=C(c1ccccc1)c1cccc(NS(C(F)F)(=O)=O)c1	-8.46	-7.25	41.29	0.312	0.282	0.745
CC(C)=CCC/C(/C)=C/COc(cc1Oc(cc2)c3cc2O)c(CC=C(C)C)c(O)c1C3=O	-8.4	-8.44	34.95	0.312	0.46	0.639
CCC(c(cc1C)cc(C)c1O[C@@H](C([O-])=O)c1ccccc1)=O	-8.49	-7.72	30.34	0.312	0.355	0.562
C[C@](CC1)([C@@H](CC2)[C@H](C[C@@H]3C(F)(F)F)[C@H]1[C@@](C)(CC1)C3=CC1=O)[C@]2(C(C)=O)O	-8.47	-8.05	25.29	0.311	0.407	0.477
**C[C@](CC1)([C@@H](CC2)[C@H](CC3)[C@H]1c(cc1)c3cc1O)[C@@]2(C#CC)O**	**-8.47**	**-8.37**	**28.01**	**0.310**	**0.457**	**0.523**
CC(C)=CCC/C(/C)=C/Cc(c(O)cc(/C=C/c1ccc(C=O)cc1)c1)c1O	-8.43	-8.66	28.13	0.309	0.502	0.525
C/C(/CC[C@@H](C(C)=C)O)=C\COc(cc1)cc2c1Oc(cc(cc1O)O)c1C2=O	-8.44	-9.11	43.91	0.309	0.572	0.789

Table S3. Interactions found by molecular docking between PTP1B$_{1-400}$ and computational hits.

		Lys_120	Ser_216	Arg_221	Ala_217	Gly_220	Tyr_46	Val_49	Phe_182	Asp_48	Asp_181	Ser_222	Ala_217	Cys_215	Ile_219				
catalytic site	OAI	X	X	X	X	X	X	X	X										
	**1a**	X	X	X	X	X	X	X		X	X				X				
	**2a**	X	X	X	X		X	X		X	X	X			X				
		X	X	X	X	X	X	X		X	X				X				
	**4a**	X	X	X	X	X		X	X	X	X		X	X	X				
	**5a**	X	X		X		X	X	X	X	X			X	X				
	**6a**	X	X	X		X	X		X	X	X			X					
	**7a**	X	X		X	X	X		X	X	X	X							
	**8a**				X		X	X	X					X					
allosteric site		**Asn_193**	**Glu_276**	**Phe_196**	**Asn_193**	**Leu_192**	**Lys_279**	**Phe_280**	**Ile_281**	**Gly_277**	**Ala_189**	**Pro_188**	**Lys_197**	**Ser_187**	**Arg_268**	**Leu_272**	**Phe_194**	**Glu_186**	**Leu_195**
	892	X	X	X	X	X	X	X	X										
	**1b**		X	X	X	X		X	X	X	X	X							X
	**2b**		X	X	X	X	X	X	X	X	X	X		X	X	X		X	
	**3b**	X	X	X		X	X	X		X	X		X			X			
	**4b**		X	X	X	X	X	X		X	X	X	X	X			X		
	**5b**	X	X	X		X	X	X	X	X	X		X						
	**7a**	X	X		X	X		X		X	X	X		X					

Chemistry

The reactions were monitored by TLC on 0.2 mm percolated silica gel 60 F254 plates (Merck, Darmstadt, Germany) and visualized by irradiation with a UV lamp. Melting points were determined on a Fisher-Johns melting point apparatus and are uncorrected. The ^{1}H-NMR and ^{13}C-NMR spectra were measured with an Agilent DD2 spectrometer (Agilent, Santa Clara, CA, USA) and a Bruker Ascend spectrometer (Bruker, Billerica, MA, USA), operating at 600 MHz and 151 MHz for ^{1}H and ^{13}C, respectively. Chemical shifts are given in parts per million relatives to tetramethylsilane (TMS, δ = 0); J values are given in Hz. The cleavage patterns are expressed as follows: s, singlet; d, doublet; t, triplet; q, quartet; dd, doublet of doublet; dt, doublet of triplet; ddd, doublet of doublets of doublets; m, multiplet; bs, broad singlet.

Synthesis of estradiol (7a)

A solution of estrone (100 mg, 0.16 mmol) in methanol (5mL) was cooled in ice bath; then, $NaBH_4$ (24 mg, 0.64 mmol) was added. The resulted mixture was stirred in ice bath for 1 h. After that, the solvent was evaporated, and the product was extracted with ethyl acetate, washed with brine, dried with anhydrous sodium sulfate, and evaporated in vacuo. The crude product was purified using column chromatography using hexanes/ethyl acetate (gradient elution from 90:10 to 50:50) to afford 91 mg (90%) of **7a** as a white solid; m.p.: 176-182 °C. The spectroscopic data matched previously reported: ^{1}H NMR (600 MHz, DMSO-d_6) δ 8.25 (d, J = 0. 9 Hz, 1H), 6.9, 6.5 and 6.4 (ddd, J = 8.4, 6.6, 0.7 Hz, 1H), 3.5 (dd, J = 8.8, 0.9 Hz, 1H), 3.0 (d, J = 8.5, Hz, 1H), 2.5 (dd, J = 8.8, 6.6, 1.0 Hz, 1H), 0.5 (dd, J = 8.4, Hz, 1H).[1,2]

References

1. Cortés-Benítez, F.; Roy, J.; Perreault, M.; Maltais, R.; Poirier, D. A- and D-Ring Structural Modifications of an Androsterone Derivative Inhibiting 17β-Hydroxysteroid Dehydrogenase Type 3: Chemical Synthesis and Structure–Activity Relationships *J. Med. Chem.* **2019**, *62* (15), 7070-7088. DOI: 10.1021/acs.jmedchem.9b00624

2. Guo J, Duclos RI Jr, Vemuri VK, Makriyannis A. The Conformations of 17β-Estradiol (E2) and 17α-Estradiol as Determined by Solution NMR. *Tetrahedron Lett*. 2010;51(27):3465-3469. doi:10.1016/j.tetlet.2010.04.077
